# Real‐time assessment of potential peak local specific absorption rate value without phase monitoring: Trigonometric maximization method for worst‐case local specific absorption rate determination

**DOI:** 10.1002/mrm.28635

**Published:** 2020-12-22

**Authors:** Ettore Flavio Meliadò, Alessandro Sbrizzi, Cornelis A. T. van den Berg, Peter R. Luijten, Alexander J. E. Raaijmakers

**Affiliations:** ^1^ Department of Radiology University Medical Center Utrecht Utrecht the Netherlands; ^2^ Computational Imaging Group for MR diagnostics & therapy Center for Image Sciences University Medical Center Utrecht Utrecht the Netherlands; ^3^ Tesla Dynamic Coils BV Zaltbommel the Netherlands; ^4^ Department of Radiotherapy Division of Imaging & Oncology University Medical Center Utrecht Utrecht the Netherlands; ^5^ Biomedical Image Analysis Department Biomedical Engineering Eindhoven University of Technology Eindhoven the Netherlands

**Keywords:** local SAR, parallel transmit, safety factor, SAR monitoring, specific absorption rate, worst‐case local SAR

## Abstract

**Purpose:**

Multi‐transmit MRI systems are typically equipped with dedicated hardware to sample the reflected/lost power in the transmit channels. After extensive calibration, the amplitude and phase of the signal at the feed of each array element can be accurately determined. However, determining the phase is more difficult and monitoring errors can lead to a hazardous peak local specific absorption rate (pSAR_10g_) underestimation. For this purpose, methods were published for online maximum potential pSAR_10g_ estimation without relying on phase monitoring, but these methods produce considerable overestimation. We present a trigonometric maximization method to determine the actual worst‐case pSAR_10g_ without any overestimation.

**Theory and Method:**

The proposed method takes advantage of the sinusoidal relation between the SAR_10g_ in each voxel and the phases of input signals, to return the maximum achievable SAR_10g_ in a few iterations. The method is applied to determine the worst‐case pSAR_10g_ for three multi‐transmit array configurations at 7T: (1) body array with eight fractionated dipoles; (2) head array with eight fractionated dipoles; (3) head array with eight rectangular loops. The obtained worst‐case pSAR_10g_ values are compared with the pSAR_10g_ values determined with a commonly used method and with a more efficient method based on reference‐phases.

**Results:**

For each voxel, the maximum achievable SAR_10g_ is determined in less than 0.1 ms. Compared to the reference‐phases‐based method, the proposed method reduces the mean overestimation of the actual pSAR_10g_ up to 52%, while never underestimating the true pSAR_10g_.

**Conclusion:**

The proposed method can widely improve the performance of parallel transmission MRI systems without phase monitoring.

## 1 INTRODUCTION

Compared to the conventional clinical systems, ultra‐high field MRI (UHF‐MRI) can achieve superior image quality.^1^, ^2^, ^3^ However, the shorter wavelength of the transmitted radiofrequency (RF) into the body of the patient results in a greater B_1_ inhomogeneity. To address this problem, several parallel transmission approaches (pTx) have been developed to modulate the amplitude and phase of the input signal.^4^, ^5^, ^6^, ^7^

This shorter wavelength also produces greater electric field (E‐Field) variability and greater power absorption by the body tissues,^8^, ^9^, ^10^ making the local specific absorption rate (SAR) limits more restrictive than the global SAR limits (as described in IEC 60601‐2‐33).^11^

Moreover, each amplitude and phase setting produces a different spatial distribution of the local SAR with the amplitude and the location of the peak value difficult to predict. Since local SAR cannot be measured during an MRI examination, it is usually evaluated with simulations. Software tools to perform online simulations using patient‐specific body models^12^ and deep learning methods for image‐based subject‐specific local SAR assessment^13^ are being developed. However, at this moment, local SAR is still evaluated by off‐line simulations using generic patient models.^14^, ^15^, ^16^, ^17^ In this approach, after domain reduction by 10g‐averaged Q‐matrices (Q_10g_)^18^, ^19^ and Virtual Observation Points (VOPs)^20^ the simulation results are stored in the MRI system to calculate a predicted peak local SAR level based on the phase and amplitude settings used at the scanner during an MR experiment.

Dedicated hardware to sample the reflected/lost power in the transmit channels is usually present in the MRI system. Assuming that the amplitude and phase of the RF waveform are properly monitored on each transmit channel and that there are no calibration errors, Q_10g_ or VOP matrices can be used to calculate the peak local SAR (pSAR_10g_) online. However, although the amplitude and phase of the signal at the feed of each array element can be accurately determined, the phase depends on the length of the cable and the actual load of the coil. These effects need to be taken into account in the calibration.

Therefore, simpler approaches to circumvent this difficulty by making conservative assumptions can be valid alternatives to advanced strategies for RF waveform monitoring.^21^, ^22^, ^23^ For example, assuming all forward power is accepted (no reflections) a more conservative pSAR_10g_ estimation is obtained (no safety risk). However, phase monitoring errors can lead to a hazardous pSAR_10g_ underestimation error.

Furthermore, in many cases it can be necessary to take into account tolerance, resolution, and malfunctions of the phase monitoring systems. Or even, some older MRI systems may just have an amplitude‐only monitoring system.

For all these reasons, methods to predict the maximum potential peak local SAR (worst‐case pSAR_10g_) for a transmit array when amplitudes are known but phases may be arbitrary will be appreciated.

Several methods are available in the literature.^24^, ^25^, ^26^, ^27^ Two of the most well‐known methods, that we refer to as total‐power‐based (TP) method and reference‐phases‐based (RP) method, were presented by Bardati and Orzada, respectively. Bardati et al^24^ showed that the amplitudes and phases which produce the maximum SAR in each location can be obtained by solving the eigenvector problem for the corresponding Q‐matrices. This method, which does not exploit the knowledge of the power distribution among the channels, actually provides the worst‐case pSAR_10g_ for the given amount of total transmit power.^26^, ^27^ Therefore, this TP method results in significant overestimation and consequent over‐conservative scanning constraints in many cases.

In order to reduce the pSAR_10g_ overestimation, Orzada et al proposed a method to approximate the maximum achievable pSAR_10g_ for a given amplitude distribution among the channels using some correction factors to prevent underestimation.^27^ However, to determine these correction factors this method solves a nested optimization problem that requires arbitrary reference phases, many iterations and random starting points. The overestimation of this method depends on the reference phases and suitable reference phases depend on the considered transmit array. With this RP method, after the correction factors have been determined, real‐time pSAR_10g_ approximation is feasible although considerable overestimation can still occur (in the investigated cases a mean overestimation up to 200% was obtained^27^).

In this work, we propose a general method to determine the maximum achievable pSAR_10g_ when only the waveform’s amplitudes in the transmit channels are known. The proposed method does not depend on arbitrary choices, and it neither overestimates nor underestimates the actual worst‐case pSAR_10g_ for a given amplitude distribution among the channels. Taking advantage of the sinusoidal relation between the local SAR in each voxel and the phases of input signals, it always returns the maximum achievable pSAR_10g_ in a few milliseconds.

The proposed method is applied to determine the worst‐case pSAR_10g_ when the power distribution over the channels is known, for three multi‐transmit array configurations at 7T: eight fractionated dipole antennas for prostate imaging^29^, ^30^; eight fractionated dipole antennas for brain imaging^31^; and eight rectangular surface coils for brain imaging.^32^ The obtained worst‐case pSAR_10g_ values are compared with the approximate maximum pSAR_10g_ values determined with the RP method^27^ and with the commonly used TP method.^24^ The results show that the proposed method can widely improve the performance of pTx MRI systems with unknown phase settings.

## 2 THEORY

The local SAR in each voxel j can be calculated from the electric field E and the properties of the tissue within the voxel (mass density ρ and electrical conductivity σ).

(1) $$SAR^{j}=\frac{\sigma^{j}}{2\rho^{j}}{\left(E^{j}\right)}^{†}E^{j}=\frac{\sigma^{j}}{2\rho^{j}}\left({\left(E_{x}^{j}\right)}^{*}E_{x}^{j}+{\left(E_{y}^{j}\right)}^{*}E_{y}^{j}+{\left(E_{y}^{z}\right)}^{*}E_{y}^{z}\right)$$

where $E_{x}$, $E_{y}$ and $E_{z}$ are the x, y and z‐components of the E‐field. For multi‐transmit systems the E‐field in each voxel is the superposition of the E‐fields transmitted by all channels.

Now we define the normalized complex electric field vectors ${\tilde{E}}_{x}$, ${\tilde{E}}_{y}$, and ${\tilde{E}}_{z}$, which contain the Cartesian E‐field components that are transmitted by each channel with a unit excitation, and the drive column vector s, which contains the complex‐valued channel amplitudes:

(2) $$\begin{array}{c} {\tilde{E}}_{x}^{j}=\left[{\tilde{E}}_{x,1}^{j},{\tilde{E}}_{x,2}^{j},\ldots ,{\tilde{E}}_{x,N_{c}}^{j}\right]\\ {\tilde{E}}_{y}^{j}=\left[{\tilde{E}}_{y,1}^{j},{\tilde{E}}_{y,2}^{j},\ldots ,{\tilde{E}}_{y,N_{c}}^{j}\right]\\ {\tilde{E}}_{z}^{j}=\left[{\tilde{E}}_{z,1}^{j},{\tilde{E}}_{z,2}^{j},\ldots ,{\tilde{E}}_{z,N_{c}}^{j}\right] \end{array}\;s=\left[\begin{array}{c} \begin{array}{c} s_{1}\\ s_{2} \end{array}\\ \begin{array}{c} \ldots \\ s_{N_{c}} \end{array} \end{array}\right]$$

where $N_{c}$ is the number of channels. The local SAR expression can be written in matrix form as follows:

(3) $$SAR^{j}=\frac{\sigma^{j}}{2\rho^{j}}s^{†}{\left({\tilde{E}}_{x}^{j}\right)}^{†}{\tilde{E}}_{x}^{j}s+\frac{\sigma^{j}}{2\rho^{j}}s^{†}{\left({\tilde{E}}_{y}^{j}\right)}^{†}{\tilde{E}}_{y}^{j}s+\frac{\sigma^{j}}{2\rho^{j}}s^{†}{\left({\tilde{E}}_{z}^{j}\right)}^{†}{\tilde{E}}_{z}^{j}s=s^{†}Q_{x}^{j}s+s^{†}Q_{y}^{j}s+s^{†}Q_{z}^{j}s.$$

The matrices $Q_{x}$, $Q_{y}$, and $Q_{z}$ have rank 1 and, hence they have only one non‐zero eigenvalue. Accordingly, the matrix $Q=Q_{x}+Q_{y}+Q_{z}$ can have at most rank 3 and three non‐zero eigenvalues, regardless of the number of channels (if at least $N_{c}\geq 3$).

When the amplitude $\left|s_{n}\right|$ and phase $\phi_{n}$ of the signal in each transmit channel are known, the application of so‐called Q‐matrices makes the local SAR calculation easy.

(4) $$SAR^{j}=s^{†}Q^{j}s.$$

For MRI, the safety limits are expressed in terms of the 10g‐averaged local SAR (IEC 60601‐2‐33^11^). Therefore, the entries of the Q‐matrices are averaged on a cube containing 10 g of tissue in order to obtain the 10g‐averaged Q‐matrices $Q_{10g}$.^19^

Subsequently, the 10g‐averaged SAR ($SAR_{10g}$) in each voxel and the peak 10g‐averaged SAR ($pSAR_{10g}$) over the whole body are calculated as follows:

(5) $$SAR_{10g}^{j}=s^{†}Q_{10g}^{j}s$$

(6) $$pSAR_{10g}=\underset{j\in \text{Body}}{\text{max}}\left(s^{†}Q_{10g}^{j}s\right).$$

Since the local SAR limits are typically defined for peak 10g‐averaged SAR levels, this study will only consider 10g‐averaged SAR levels. To avoid symbols densely packed with subscripts and superscripts, the subscript “10g” is from this point onwards omitted. Whenever SAR or pSAR are mentioned, it actually refers to, respectively, SAR_10g_ and pSAR_10g_.

As already mentioned in the introduction, although almost every pTx system monitors the amplitude and phase of the signals being emitted, for example, by means of bi‐directional couplers,^23^ without extensive calibration, a deviating loading condition of a transmit array element may result in a hazardous deviating phase in comparison to simulated field distributions (pSAR underestimation error). Moreover, in many cases, it can be necessary to take into account tolerance, resolution, and malfunctions of the phase monitoring systems.

Therefore, in order to ensure patient safety, it can be useful to determine the maximum pSAR that can be reached by a given amplitude set (without phase information).

A method commonly used for this purpose does not exploit the knowledge of the power distribution among the channels.^24^ With this method, based on the min‐max theorem in linear algebra, the maximum SAR value of the quadratic form $s^{†}Q_{10g}s$, for any possible set of phases with a given total power ${∥s∥}_{2}^{2}=P_{\mathrm{Tot}}$, is determined by multiplying the largest eigenvalue $\lambda_{\max}$ of $Q_{10g}$ by the total power transmitted by all channels together.

(7) $$\lambda_{\text{max}}^{j}{∥s∥}_{2}^{2}=\underset{s,{∥s∥}_{2}^{2}=P_{\mathrm{Tot}}}{\text{max}}\left(s^{†}Q_{10g}^{j}s\right)$$

This TP method is generally used to define an upper‐bound for the maximum achievable pSAR ($pSAR_{\mathrm{TP}}$) when the power in each transmit channel is known.^24^, ^25^, ^26^, ^27^

(8) $$pSAR_{\mathrm{TP}}=\underset{j\in \text{Body}}{\text{max}}\left(\lambda_{\text{max}}^{j}\right){∥s∥}_{2}^{2}$$

This upper‐bound is only reached for a drive vector s with magnitude of the components equal or proportional to the magnitude of the components of the eigenvector $v_{\max}$ associated to the largest eigenvalue. Therefore, Equation (8) often results in an overly conservative overestimation of the maximum achievable pSAR value. Indeed, as already mentioned, in most cases only the phase $\phi_{n}$ of the signal in each transmit channel is unknown and the maximum achievable pSAR value is given the known amplitudes set is much lower.^27^

To reduce this excessive overestimation, a method which exploits the knowledge of the signal amplitude in each transmit channel to approximate the maximum achievable pSAR has recently been published by Orzada et al.^27^ This RP method solves a nested optimization problem to obtain K correction factors $\zeta_{k}$ for K reference sets of phases $P_{k}={\left[e^{i\phi_{1,k}},e^{i\phi_{2,k}},\ldots ,e^{i\phi_{N_{c},k}}\right]}^{T}$. Because this optimization can run into local optima, multiple random starting points were used. Subsequently, these correction factors are used to approximate a conservative upper‐bound for the maximum achievable pSAR based on reference phases ($pSAR_{\mathrm{RP}}$).

(9) $$pSAR_{\mathrm{RP}}=\underset{k\in P}{\text{min}}\left(\zeta_{k}\;\cdot \;\underset{j\in \text{Body}}{\text{max}}\left(w_{k}^{†}Q_{10g}^{j}w_{k}\right)\right)$$

where $w_{k}={\left[\left|s_{1}\right|e^{i\phi_{1,k}},\;\left|s_{2}\right|e^{i\phi_{2,k}},\ldots ,\left|s_{\mathrm{Ne}}\right|e^{i\phi_{N_{c},k}}\right]}^{T}$ is a column vector with magnitudes equal to the drive vector magnitudes and phases equal to the *k*‐th set of reference phases. When the obtained upper‐bound is higher than the maximum achievable pSAR with the same total input power, $pSAR_{\mathrm{RP}}$ is set equal to $pSAR_{\mathrm{TP}}$. With this method, the overestimation can be significantly reduced compared to the TP method (up to 50%), although large overestimation could still occur (up to 200%).^27^ A complicating factor of this RP method is that depends on the choice of the reference phase sets P. Suitable reference phases depend on the considered transmit array. Thus, this dependency on the reference phase sets make its performance variable.

In this work, we propose an alternative method that allows fast calculation of the maximum achievable pSAR without any over‐ or under‐estimation. For this purpose, the local SAR equation is reformulated to bring out the sinusoidal relation with the phases of the drive vector s. In fact, Equation (5)) can be reformulated as follow:

(10) $$\begin{array}{c} SAR^{j}=\sum_{n=1}^{N_{c}}\sum_{m=1}^{N_{c}}\left|s_{n}\right|\left|Q_{n,m}^{j}\right|\left|s_{m}\right|e^{i\left(‐\phi_{n}+\vartheta_{n,m}^{j}+\phi_{m}\right)}=\sum_{n\;=\;1}^{N_{c}}\left|s_{n}\right|\left|Q_{n,n}^{j}\right|\left|s_{n}\right|\\ +\sum_{n=1}^{N_{c}}\sum_{m=n+1}^{N_{c}}\left(\left|s_{n}\right|\left|Q_{n,m}^{j}\right|\left|s_{m}\right|e^{i\left(‐\phi_{n}+\vartheta_{n,m}^{j}+\phi_{m}\right)}+\left|s_{m}\right|\left|Q_{m,n}^{j}\right|\left|s_{n}\right|e^{i\left(‐\phi_{n}+\vartheta_{n,m}^{j}+\phi_{m}\right)}\right) \end{array}$$

where $\left|s_{n}\right|$ and $\phi_{n}$ are, respectively, the amplitude and phase of the input signal in each channel, and $\left|Q_{m,n}^{j}\right|$ and $\vartheta_{n,m}^{j}$ are, respectively, the amplitude and phase of the $Q_{10g}$ matrix entries.

Note that $Q_{10g}$ is Hermitian, that is, $\left|Q_{m,n}^{j}\right|=\left|Q_{n,m}^{j}\right|$ and $\vartheta_{m,n}^{j}=‐\vartheta_{n,m}^{j}$. Then we can write:

(11) $$SAR^{j}=\sum_{n=1}^{N_{c}}\left|s_{n}\right|\left|Q_{n,n}^{j}\right|\left|s_{n}\right|+\sum_{n=1}^{N_{c}}\sum_{m=n+1}^{N_{c}}\left|s_{n}\right|\left|Q_{n,m}^{j}\right|\left|s_{m}\right|\left(e^{i\left(‐\phi_{n}+\vartheta_{n,m}^{j}+\phi_{m}\right)}+e^{‐i\left(‐\phi_{n}+\vartheta_{n,m}^{j}+\phi_{m}\right)}\right)$$

Then, using Euler's formula

(12) $$SAR^{j}=\sum_{n=1}^{N_{c}}\left|s_{n}\right|\left|Q_{n,n}^{j}\right|\left|s_{n}\right|+\sum_{n=1}^{N_{c}}\sum_{m=n+1}^{N_{c}}2\left|s_{n}\right|\left|Q_{n,m}^{j}\right|\left|s_{m}\right|\text{cos}\left(‐\phi_{n}+\vartheta_{n,m}^{j}+\phi_{m}\right)$$

**FIGURE 1:**
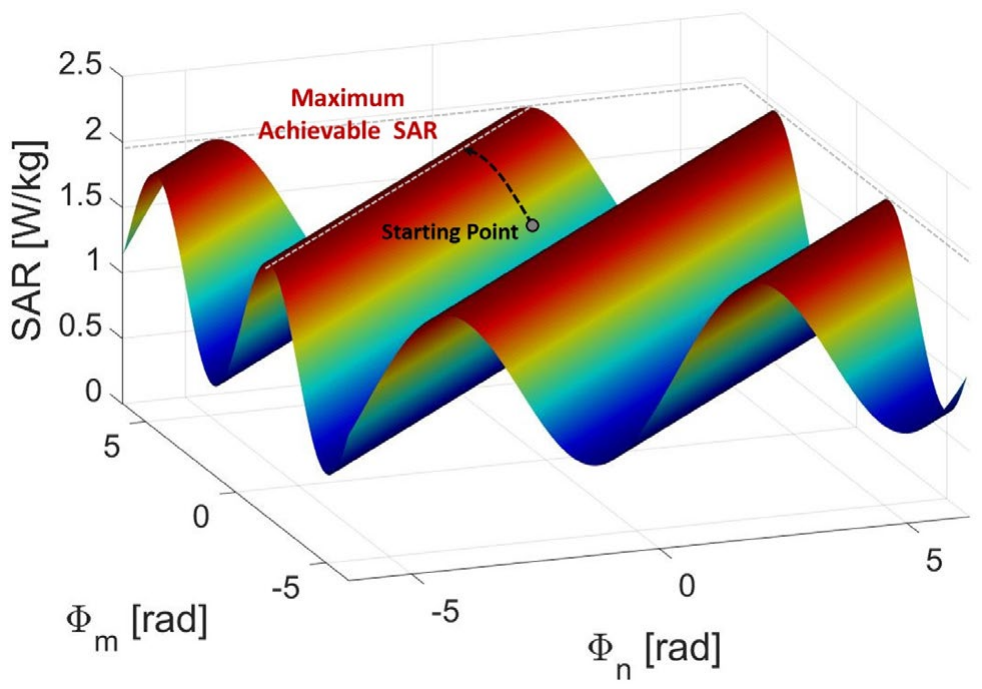
Sinusoidal relation between SAR in a voxel and the phase of signal in the transmit channels *n* and *m*. The local maximum achievable SAR is a periodic repetition of the global maximum, and it can be achieved from any starting point following the direction of the maximum ascent

The second phase‐dependent term of this expression is a linear combination of cosine functions, which results in a sinusoidal SAR variation. Figure 1 highlights this sinusoidal relation between SAR and drive vector phases. In particular, it shows an example of how the SAR varies in a voxel when only the phase of two input signals change (Equation 12).

Therefore, each local maximum is a periodic repetition of the global maximum. Thus, finding a local maximum is equivalent to finding a global maximum.

With a number of channels larger than two, the sinusoidal SAR variation is more difficult to view, however it follows a similar trend. The local maximum values are on multi‐dimensional parallel “straight” lines. (Supporting Information Figure S1, which is available online).

Thus, for a given amplitude set $\left|s_{n}\right|$, the desired phase set $\phi_{\mathrm{Max}}$ that provides the global maximum (or also the minimum) SAR in each voxel can be determined by solving the corresponding stationary points equations:

(13) $$\nabla SAR^{j}\left(\phi \right)=0$$

This is a system of equations where the $N_{c}$ gradient components need to be zero:

for $l=1,2,\ldots ,N_{c}$.

(14) $$\nabla_{\phi_{l}}SAR^{j}=\;\sum_{m=l+1}^{N_{c}}2\left|s_{l}\right|\left|Q_{l,m}^{j}\right|\left|s_{m}\right|\text{sin}\left(‐\phi_{l}+\vartheta_{l,m}^{j}+\phi_{m}\right)‐\sum_{n=1}^{l‐1}2\left|s_{n}\right|\left|Q_{n,l}^{j}\right|\left|s_{l}\right|\text{sin}\left(‐\phi_{n}+\vartheta_{n,l}^{j}+\phi_{l}\right)=0.$$

The solutions of this system of equations can produce a maximum or a minimum SAR. Since we want to determine the maximum SAR, we are interested in the solutions where the Hessian matrix is negative‐definite (ie, a concave point of the SAR Equation 12).

Then, using the drive vector consisting of the known amplitudes and the determined phase set, $s_{\mathrm{Max}}^{j}=\left|s\right|e^{i\phi_{\mathrm{Max}}^{j}}$, the maximum achievable SAR for each voxel can be calculated. Thus, the actual worst‐case pSAR ($pSAR_{\mathrm{WoC}}$) for the given amplitude set can be determined as follows.

(15) $$pSAR_{\mathrm{WoC}}=\underset{j\in \text{Body}}{\text{max}}\left(s_{\text{Max}}^{j†}Q_{10g}^{j}s_{\text{Max}}^{j}\right).$$

However, we do notsolve the previous system of equations analytically. The phase set that produces the maximum SAR can be obtained through a fixed‐point iterations scheme for the solution of Equation (14) that will be elaborated in the methods section.

The proposed method, based on trigonometric properties of the SAR equation, does not overestimate (or underestimate) the maximum achievable pSAR, but it always returns the actual worst‐case pSAR with the required precision.

### 2.1 Further analysis: Peak local SAR approximations

In the following part of the theory, a further analysis is presented where an upper‐bound and a lower‐bound to the actual worst‐case pSAR are introduced. These bounds can be calculated directly from the Q‐matrix entries and therefore do not require a numerical optimization.^28^

For each voxel, the Q‐matrix Q could have at most three non‐zero eigenvalues because the electric field vector of each transmit element has three Cartesian components. When only one E‐field component is present (or one E‐field component is dominant, eg, the z‐component) Q will have only one non‐zero or dominant eigenvalue. In this case, the phases of the components of the eigenvector corresponding to the non‐zero eigenvalue cancel the phase of each Q‐matrix entry, that is, $\left(‐\phi_{n}+\vartheta_{n,m}+\phi_{m}\right)=0$, for each n,m in Equation (10). In this case, worst‐case pSAR corresponds to perfect constructive summing/interference of the E‐fields of each transmit array element (see Supporting Information Appendix S1). The resulting maximum SAR is equal to the sum of the magnitude of all entries of Q. Note that even when three Cartesian components are present, but the E‐fields transmitted by all elements have the same direction (ie, E‐fields transmitted by all elements are parallel), Q has only one non‐zero eigenvalue and the same applies (indeed, a rotated frame of reference XYZ′ exists where all E‐fields are in z′‐direction; thus, the same argument as above applies).

Assuming negligibly small variations of the electric properties and the E‐field distributions in the region that contains 10g of tissue, these considerations could be applied also to 10g‐averaged Q‐matrices $Q_{10g}$. Thus, when the $Q_{10g}$ matrices have only one eigenvalue, the maximum pSAR actually achievable is:

(16) $$pSAR_{\mathrm{WoC}}=\underset{j\in \text{Body}}{\text{max}}\left(\sum_{n=1}^{N_{c}}\sum_{m=1}^{N_{c}}\left|s_{n}\right|\left|Q_{n,m}^{j}\right|\left|s_{m}\right|e^{i\left(‐\psi_{n^{j}}+\vartheta_{n,m}^{j}+\psi_{m^{j}}\right)}\right)=\underset{j\in \text{Body}}{\text{max}}\left(\sum_{n=1}^{N_{c}}\sum_{m=1}^{N_{c}}\left|s_{n}\right|\left|Q_{n,m}^{j}\right|\left|s_{m}\right|\right)$$

where $\psi_{n^{j}}$ are the phases of the components of the eigenvector $v^{j}$ associate to the eigenvalue $\lambda^{j}$ of the matrix $Q_{10g}^{j}$.

When the E‐field presents more than one Cartesian component, that is, $Q_{10g}^{j}$ has more non‐zero eigenvalues, the phases of the components of the eigenvector associated to the maximum eigenvalue produces a lower‐bound for the worst‐case pSAR ($pSAR_{\mathrm{LB}}$).

(17) $$pSAR_{\mathrm{WoC}}\geq pSAR_{\mathrm{LB}}=\underset{j\in \text{Body}}{\text{max}}\left(\sum_{n=1}^{N_{c}}\sum_{m=1}^{N_{c}}\left|s_{n}\right|\left|Q_{n,m}^{j}\right|\left|s_{m}\right|e^{i\left(‐\psi_{n^{j}}+\vartheta_{n,m}^{j}+\psi_{m^{j}}\right)}\right)$$

whereas, the sum of only magnitude terms produces an upper‐bound for the worst‐case pSAR ($pSAR_{\mathrm{UB}}$).

(18) $$pSAR_{\mathrm{WoC}}\leq pSAR_{\mathrm{UB}}=\underset{j\in \text{Body}}{\text{max}}\left(\sum_{n=1}^{N_{c}}\sum_{m=1}^{N_{c}}\left|s_{n}\right|\left|Q_{n,m}^{j}\right|\left|s_{m}\right|\right).$$

It is worth noting that RF coil arrays for MRI are usually designed to produce an efficient circular polarization of the magnetic field on the x‐y plane ($B_{1x}$, $B_{1y}$‐components). This results in a dominant z‐component of the transmitted E‐field. For example, dipole antenna arrays have only one dominant Cartesian E‐field component (the $E_{z}$‐component).

Moreover, with many RF coil array configurations, the maximum achievable pSAR value is often located near the array where the E‐field transmitted by one element is dominant^33^ or in a region where the E‐fields of all elements have a similar direction (where their constructive interference is more effective). Therefore, although it cannot be guaranteed, one dominant eigenvalue generally exists in those regions where the maximum pSAR is located. Then, the actual worst‐case pSAR could be approximated with one of the two proposed methods.

## 3 METHODS

### 3.1 Trigonometric maximization method for worst‐case local SAR determination

The phase set that produces the worst‐case pSAR can be obtained through a fixed‐point iterations scheme for the solution of the stationary Equation (14).

Since, the solutions of the stationary equations can produce a maximum or a minimum SAR, we define an ad‐hoc iteration function $\phi =G\left(\phi \right)$ which always converges to the phase set that produces the maximum SAR value.

To define this $G\left(\phi \right)$ function we transform the system of stationary equations $\nabla SAR\left(\phi \right)=0$. Starting from the system of Equation (14), we apply the addition formulas for sine

for $l=1,2,\ldots ,N_{c}$.

(19) $$\begin{array}{c} \nabla_{\phi_{l}}SAR=\sum_{m=l+1}^{N_{c}}2\left|s_{l}\right|\left|Q_{l,m}\right|\left|s_{m}\right|\text{sin}\left(\vartheta_{l,m}\right)\text{cos}\left(‐\phi_{l}+\phi_{m}\right)+2\left|s_{l}\right|\left|Q_{l,m}\right|\left|s_{m}\right|\text{cos}\left(\vartheta_{l,m}\right)\text{sin}\left(‐\phi_{l}+\phi_{m}\right)\\ ‐\sum_{n=1}^{l‐1}2\left|s_{n}\right|\left|Q_{n,l}\right|\left|s_{l}\right|\text{sin}\left(\vartheta_{n,l}\right)\text{cos}\left(‐\phi_{n}+\phi_{l}\right)+2\left|s_{n}\right|\left|Q_{n,l}\right|\left|s_{l}\right|\text{cos}\left(\vartheta_{n,l}\right)\text{sin}\left(‐\phi_{n}+\phi_{l}\right)=0. \end{array}$$

Defining $Ax_{n,l}$, $Ay_{n,l}$, $Ax_{l,m}$ and $Ay_{l,m}$

(20) $$\begin{array}{c} Ax_{n,l}=2\left|s_{n}\right|\left|Q_{n,l}\right|\left|s_{l}\right|\text{cos}\left(\vartheta_{n,l}\right)\;Ax_{l,m}=2\left|s_{l}\right|\left|Q_{l,m}\right|\left|s_{m}\right|\text{cos}\left(\vartheta_{l,m}\right)\\ Ay_{n,l}=2\left|s_{n}\right|\left|Q_{n,l}\right|\left|s_{l}\right|\text{sin}\left(\vartheta_{n,l}\right)\;Ay_{l,m}=2\left|s_{l}\right|\left|Q_{l,m}\right|\left|s_{m}\right|\text{sin}\left(\vartheta_{l,m}\right) \end{array}$$

and using again the addition and subtraction formulas for sine and cosine, a closed expression can be found for $\phi_{l}$, which would then be our iteration function $G\left(\phi \right)$ (detailed derivation in Appendix 1):

(21) $$\phi_{l}=\text{arctan}\left(\frac{\left(\sum_{m=l+1}^{N_{c}}Ay_{l,m}\text{cos}\left(\phi_{m}\right)+Ax_{l,m}\text{sin}\left(\phi_{m}\right)‐\sum_{n=1}^{l‐1}Ay_{n,l}\text{cos}\left(\phi_{n}\right)‐Ax_{n,l}\text{sin}\left(\phi_{n}\right)\right)}{‐\left(\sum_{m=l+1}^{N_{c}}Ay_{l,m}\text{sin}\left(\phi_{m}\right)‐Ax_{l,m}\text{cos}\left(\phi_{m}\right)‐\sum_{n=1}^{l‐1}Ay_{n,l}\text{sin}\left(\phi_{n}\right)+Ax_{n,l}\text{cos}\left(\phi_{n}\right)\right)}\right)$$

Equation (21) represents the *l*‐th function of the new system of equations defined to iteratively solve the equivalent system of Equation (14). The arctangent function is defined as the inverse function of the tangent within the range −π/2 to π/2. However, the period of SAR Equation (12) is 2π. To allow convergence starting from any point, we use the “two‐argument arctangent” function instead of the arctangent function. The resulting expression is the iteration function $G\left(\phi \right)$:

(22) $$\begin{array}{c} Y_{l}=\left(\sum_{m=l+1}^{N_{c}}Ay_{l,m}\text{cos}\left(\phi_{m}\right)+Ax_{l,m}\text{sin}\left(\phi_{m}\right)‐\sum_{n=1}^{l‐1}Ay_{n,l}\text{cos}\left(\phi_{n}\right)‐Ax_{n,l}\text{sin}\left(\phi_{n}\right)\right)\\ X_{l}=‐\left(\sum_{m=l+1}^{N_{c}}Ay_{l,m}\text{sin}\left(\phi_{m}\right)‐Ax_{l,m}\text{cos}\left(\phi_{m}\right)‐\sum_{n=1}^{l‐1}Ay_{n,l}\text{sin}\left(\phi_{n}\right)+Ax_{n,l}\text{cos}\left(\phi_{n}\right)\right) \end{array}$$

(23) $$G_{l}:=\text{arctan}2\left(Y_{l},X_{l}\right).$$

The implemented iterative algorithm to determine the phase set $\phi_{\mathrm{TM}}$ that maximized the SAR for each voxel is briefly described below (ε is the required accuracy on the maximum local SAR and can be so small such that it produces no significant underestimation, eg, $\varepsilon =10^{‐6}$).

**Table:** 

**Algorithm (fixed point iterations)**
**Initial Phase Set** $\phi^{0}=\left[0;0;\ldots ;0\right]$, k=1
**While** $SAR\left(\phi^{k}\right)‐\text{SAR}\left(\phi^{k‐1}\right)\leq \varepsilon$
**For** l=1 to $l=N_{c}$
$Y_{l}^{k}=\left(\sum_{m=l+1}^{N_{c}}Ay_{l,m}\text{cos}\left(\phi_{m}^{k‐1}\right)+Ax_{l,m}\text{sin}\left(\phi_{m}^{k‐1}\right)‐\sum_{n=1}^{l‐1}Ay_{n,l}\text{cos}\left(\phi_{n}^{k‐1}\right)‐Ax_{n,l}\text{sin}\left(\phi_{n}^{k‐1}\right)\right)$
$X_{l}^{k}=‐\left(\sum_{m=l+1}^{N_{c}}Ay_{l,m}\text{sin}\left(\phi_{m}^{k‐1}\right)‐Ax_{l,m}\text{cos}\left(\phi_{m}^{k‐1}\right)‐\sum_{n=1}^{l‐1}Ay_{n,l}\text{sin}\left(\phi_{n}^{k‐1}\right)+Ax_{n,l}\text{cos}\left(\phi_{n}^{k‐1}\right)\right)$
$\phi_{l}^{k}=a\text{tan}2\left(Y_{l}^{k},X_{l}^{k}\right)$
**End** k=k+1 **End**

The iterative optimization algorithm described above is implemented in Matlab (MATLAB, The MathWorks, Inc., Natick, MA) and included in the Supporting Information.

Then, using the drive vector consisting of the known amplitudes and the obtained phase set for each voxel ($s_{\mathrm{TM}}^{j}=\left|s\right|e^{i\phi_{\mathrm{TM}}^{j}}$), the maximum achievable pSAR can be calculated with the required precision ($pSAR_{\mathrm{TM}}$).

(24) $$pSAR_{\mathrm{TM}}=\underset{j\in \text{Body}}{\text{max}}\left(s_{\mathrm{TM}}^{j†}Q_{10g}^{j}s_{\mathrm{TM}}^{j}\right).$$

### 3.2 Worst‐case peak local SAR determination with three transmit array configurations

**FIGURE 2:**
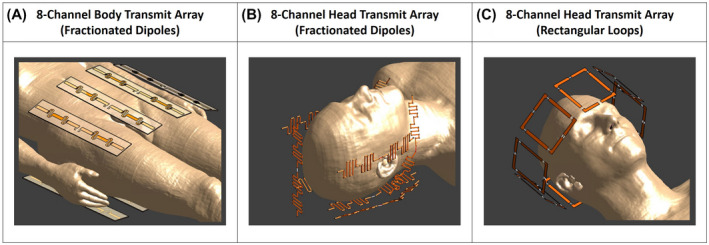
The investigated transmit array setups: body transmit array for prostate imaging composed of eight fractionated dipoles (A), head transmit array composed of eight fractionated dipoles (B), head transmit array composed of eight rectangular loops (C)

The proposed method is applied to determine the worst‐case pSAR for three different transmit arrays at 7T. A body transmit array for prostate imaging composed of eight fractionated dipoles,^29^, ^30^ and two head transmit arrays, one composed of eight fractionated dipoles^31^ and the other composed of eight rectangular loops^32^ (Figure 2).

For each transmit array, finite‐difference time‐domain (FDTD) simulations are performed (Sim4Life, ZMT, Zürich, Switzerland) with the commonly used patient model “Duke” of the Virtual Family with 77 tissues.^14^, ^15^ The results are processed to obtain Q‐matrices^18^ and 10g‐averaged Q‐matrices.^19^ Subsequently, the VOPs^20^ are determined with an allowed maximum overestimation of 5% of the maximum eigenvalues over all $Q_{10g}$ matrices.

Using the obtained VOP set, for each MRI examination scenario, the worst‐case pSAR value is determined by the proposed trigonometric maximization (TM) method with 1,000,000 random drive vectors normalized to 1W total input power. The determined worst‐case pSAR value is compared to the approximated maximum achievable pSAR value obtained with the recently published method based on reference phases^27^ and to the maximum achievable pSAR value considering the total power transmitted by all channels together (without exploiting the knowledge of the power distribution among the channels, a less efficient but more common method).

The VOP set of each considered scenario is also used to estimate the actual pSAR value for each drive vector in order to assess the mean overestimation and the reliability of the method (it should never show underestimation).

## 4 RESULTS

The VOP set was calculated for each transmit array setup (maximum overestimation of 5%), resulting in: 777 VOPs for the body transmit array with eight fractionated dipoles (A), 4418 VOPs for the head transmit array with eight fractionated dipoles (B), and 2578 VOPs for the head transmit arrays with eight rectangular loops (C).

For each transmit array setup and for each random drive vector normalized to 1W total input power, the VOP set was used to estimate: the worst‐case pSAR value with the proposed TM method ($pSAR_{\mathrm{TM}}$); the approximation of the maximum achievable pSAR with the RP method^27^ ($pSAR_{\mathrm{RP}}$); the highest physically possible pSAR for 1W total input power with the TP method ($pSAR_{\mathrm{TP}}$). Furthermore, each VOP set was also used to estimate the actual pSAR for each drive vector (pSAR).

**FIGURE 3:**
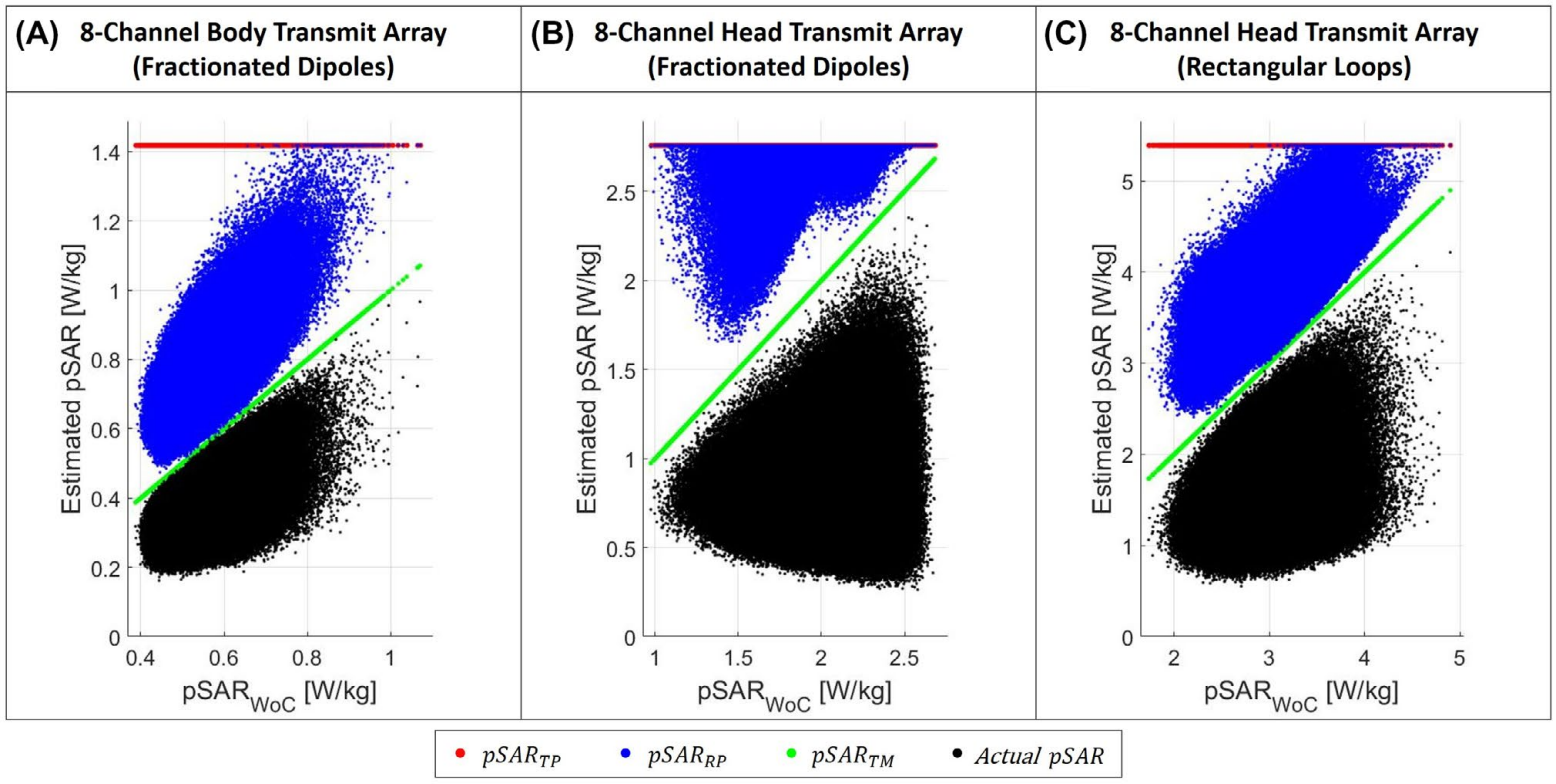
Scatter plots of estimated worst‐case pSAR versus actual worst‐case pSAR for a collection of 1,000,000 random phase‐amplitude settings. Results are plotted for body transmit array composed of eight fractionated dipoles (A), head transmit array composed of eight fractionated dipoles (B), head transmit array composed of eight rectangular loops (C). The green dots are the estimated worst‐case pSAR values with the proposed TM method (pSAR_TM_). The blue dots are the approximated maximum achievable pSAR values obtained with RP method (pSAR_RP_). The red dots are the maximum achievable pSAR values considering the total power transmitted (1W) with the commonly used TP method (pSAR_TP_). The black dots are the actual pSAR values considering amplitudes and phases settings

Figure 3 shows the scatter plots of estimated pSAR versus actual worst‐case pSAR for one million points each. The green dots are the estimated worst‐case $pSAR_{\mathrm{TM}}$ values with the proposed TM method. Because we assume $pSAR_{\mathrm{TM}}=pSAR_{\mathrm{WoC}}$, they follow the diagonal. The red dots are the estimated highest $pSAR_{\mathrm{TP}}$ values for the given total input power. Therefore, they follow a horizontal line because each drive vector has the same total power (1W). The blue dots scattered between the green and red “dot lines” are the estimated worst‐case pSAR values with the RP method ($pSAR_{\mathrm{RP}}$). Some of these solutions are “touching” the $pSAR_{\mathrm{TM}}$ as expected but most are above.

**FIGURE 4:**
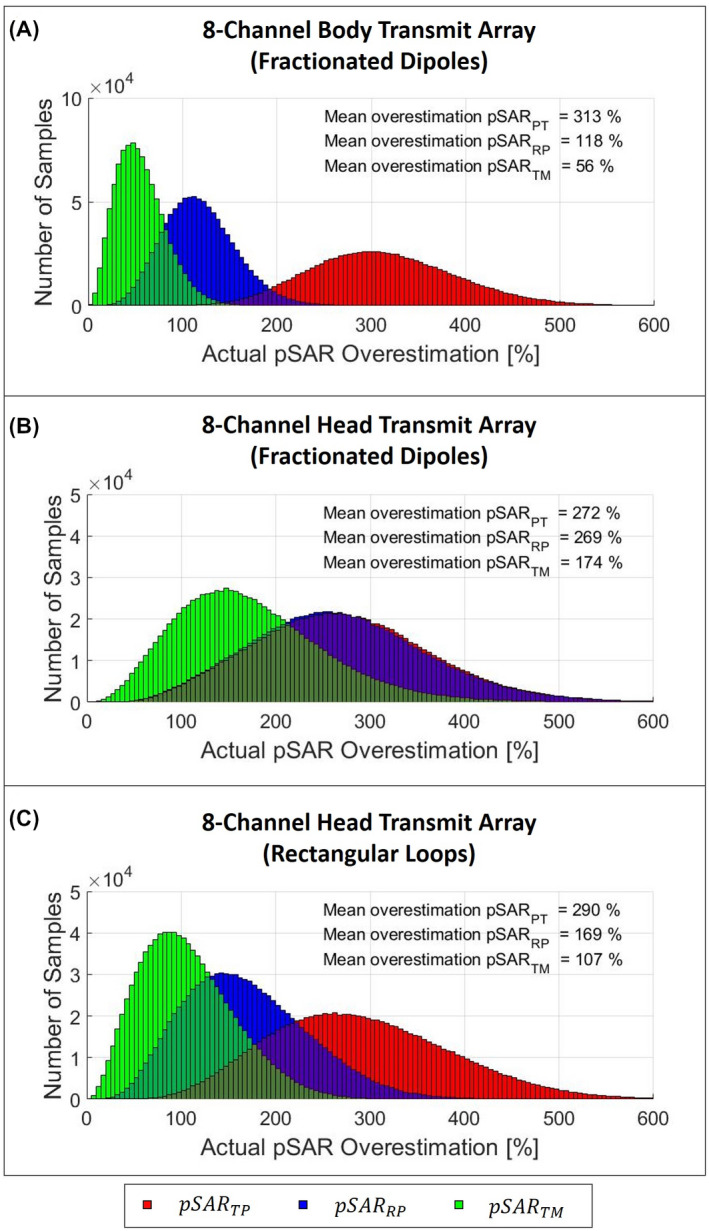
Histogram of the actual pSAR overestimation for each investigated transmit array setup: body transmit array composed of eight fractionated dipoles (A), head transmit array composed of eight fractionated dipoles (B), head transmit array composed of eight rectangular loops (C). The green blocks show the overestimation distribution with the proposed TM method (pSAR_TM_). The blue blocks show the overestimation distribution with RP method (pSAR_RP_). The red blocks show the overestimation distribution with the commonly used TP method (pSAR_TP_)

In Figure 3 are also reported the actual pSAR values. The black dots are the actual pSAR values for every drive vector (considering amplitudes and phases). As expected, the green dot line presents an almost perfect delineation of the maximum‐possible pSAR value. None of the black points are above the green points, which confirms that the proposed method never produces underestimation errors. These results validate our proposition that $pSAR_{\mathrm{WoC}}$ and $pSAR_{\mathrm{TM}}$ are in fact identical. In addition, none of the blue (and red) points are below green points showing that the trigonometric method always produces the lowest overestimation of the actual pSAR. This overestimation reduction is also quantitatively described in Figure 4. This figure shows, for each investigated transmit array setup, the histogram of the overestimation of the actual pSAR values of each considered estimation method. The histograms clearly show the benefits of the proposed method (green blocks) compared to existing methods, with a mean overestimation reduction of 52% for the body transmit array, 35% for the head transmit array with fractionated dipoles, and 37% for the head transmit array with rectangular loops.

These results show that the presented TM method is not only better than previously published methods. It is also optimal because it always finds the exact value of the worst‐case pSAR for that combination of amplitudes with the required precision in a few milliseconds (Table 1).

**TABLE 1:** Required calculation time for each investigated transmit array setup and each method to estimate the potential pSAR value without phase monitoring (Workstation: Intel(R) Core(TM) i5‐4590 CPU @ 3.30GHz – RAM 16.0 GB)

Transmit array	Number of VOPs	Required calculation time [ms]				
		pSAR_TP_	pSAR_RP_	pSAR_TM_	pSAR_LB_	pSAR_UB_
Body array (8 Fractionated dipoles)	777	0.02	2.3	18	0.3	0.5
Head array (8 Fractionated dipoles)	4418	0.02	18	107	2.2	3.5
Head array (8 Rectangular loops)	2578	0.02	11	61	1.4	2.2

**FIGURE 5:**
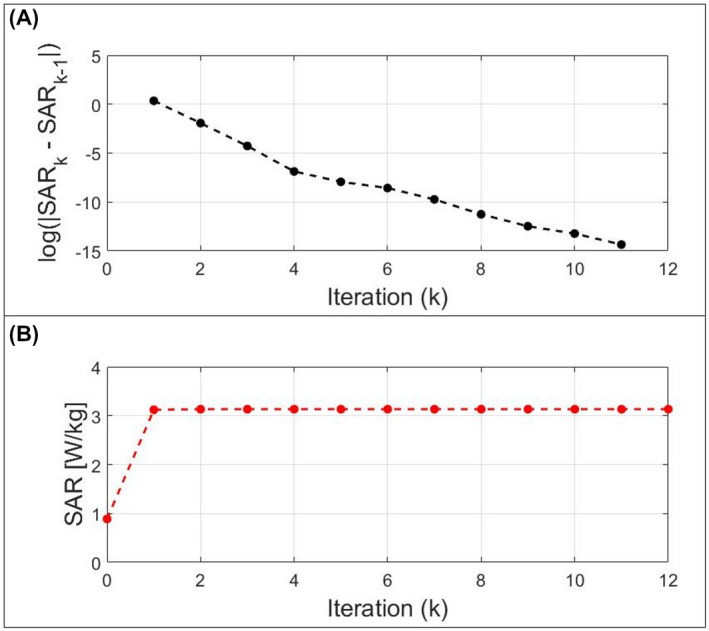
Example of the exponential convergence of the implemented TM method. A, Logarithm of the SAR estimation error for each iteration. B, Estimated SAR for each iteration

Figure 5 highlights the exponential convergence of the implemented TM method. It achieves a residual error lower than 10^−16^ in around 10 iterations and provides the maximum SAR with an accuracy of 10^−3^ after just one iteration.

**FIGURE 6:**
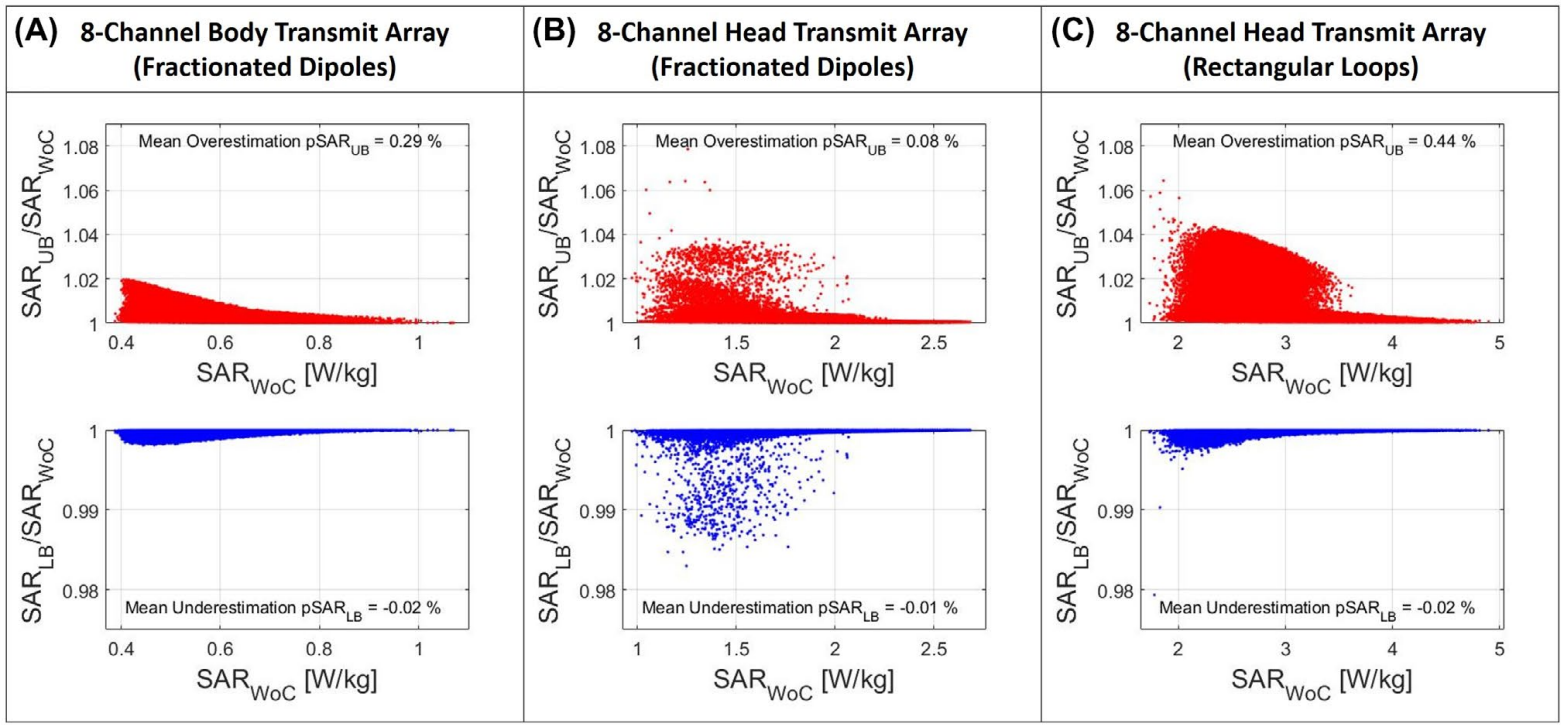
Scatter plot of the ratio pSAR_UB_/pSAR_WoC_ versus actual worst‐case pSAR_WoC_ (red dots), and the scatter plot of ratio pSAR_LB_/pSAR_WoC_ versus actual worst‐case pSAR_WoC_ (blue dots), for each investigated transmit array setup: body transmit array composed of eight fractionated dipoles (A), head transmit array composed of eight fractionated dipoles (B), head transmit array composed of eight rectangular loops (C)

The accuracy of the two proposed approximation methods is shown in Figure 6. For each investigated array setup, it shows the scatter plot of the ratio $pSAR_{\mathrm{UB}}/pSAR_{\mathrm{WoC}}$ versus $pSAR_{\mathrm{WoC}}$, and the scatter plot of ratio $pSAR_{\mathrm{LB}}/pSAR_{\mathrm{WoC}}$ versus $pSAR_{\mathrm{WoC}}$. Compared to the actual worst‐case pSAR, both approximation methods show a small mean estimation error (the upper‐bound $pSAR_{\mathrm{UB}}$ shows a mean overestimation error from 0.1% to 0.4%, the lower‐bound $pSAR_{\mathrm{LB}}$ shows a mean underestimation error from 0.01% to 0.02%) and a very short calculation time, Table 1 (between 0.3 ms and 3 ms for the whole VOP sets, almost two orders of magnitude faster). However, overestimation errors up to 8% ($pSAR_{\mathrm{UB}}$) and underestimation errors up to 2% ($pSAR_{\mathrm{LB}}$) can rarely occur.

## 5 DISCUSSION

MRI systems are typically able to sample the amplitude and phase of the transmitted RF waveforms. However, although the amplitude and phase at the coil can be accurately determined, without extensive calibration, phase monitoring errors can lead to a hazardous pSAR underestimation error. The existing methods for online assessment of potential pSAR value without phase monitoring produce considerable overestimation of the actual worst‐case pSAR. In this work a TM method for worst‐case pSAR determination is presented. This maximization method takes advantage of the sinusoidal relation between the pSAR in each voxel and the phases of input signals, to return the maximum achievable SAR for each voxel in less than 0.1 ms.

For a given amplitude set, the implemented algorithm determines for each voxel (ie, for each Q_10g_ matrix), the phase set which produces the maximum achievable SAR in very few interactions (exponential convergence, Figure 5). This allows determining the actual worst‐case pSAR for a typical VOP set in a few milliseconds (Table 1).

Since the proposed method is inherently parallel, in the case of 3D geometries with millions of voxels, the overall computation time required can also be significantly reduced using graphics processing units.

Compared to the RP method presented by Orzada et al,^27^ the proposed method reduces the mean overestimation of the actual pSAR by 35% to 52%. A large overestimation can still occur (eg, 174%, Figure 4B). However, this overestimation cannot be reduced because it is not the result of a conservative estimation but it only is due to phase uncertainties. In particular, for the head transmit array with fractionated dipoles, with the same amplitude set, the range of possible pSAR values is very large. Therefore, the actual worst‐case pSAR values (Figure 3B, green dots) are often much higher than the actual pSAR values (black dots). Indeed, the observed pSAR values range from 0.3 W\kg to 2.3 W\kg, with a mean value of about 0.8 W/kg. This also explains why the commonly used TP method ($pSAR_{\mathrm{TP}}=2.8W/kg$) produces a mean overestimation of about 272% for this array setup. This great pSAR variability also produces large correction factors (Supporting Information Table S1) and the large overestimation with the latest published RP method (269%), which, probably due to unsuitable reference phases for this array setup, rarely determines pSAR values lower than the commonly used TP method, and fails to reduce overestimation.

For this transmit array setup, the large pSAR variability is probably due to a reduced distance between the dipoles of the array, which results in pSAR values produced by the interference of E fields of similar amplitude leading to a higher potential modulation depth, that is, relatively large worst‐case pSAR compared to the mean pSAR.

Whereas for array setups where the pSAR values are mainly due to just one transmit element^33^ (eg, body array with fractionated dipoles, where they are typically located in the region immediately under the array element), this pSAR variability is much smaller (Figure 3A).

It is worth noting that, since the investigated body array for prostate imaging is composed of parallel fractionated dipoles, it shows a very dominant z‐component of the transmitted E‐field (Supporting Information Figure S2). Whereas, in many head regions (eg, neck, cheeks, forehead, etc.), no strongly dominant components are observed with the head array with oblique fractionated dipoles and rectangular loops (Supporting Information Figures S3 and S4).

Since the fixed point in (−π, π) is not unique (fixed points for maximum and minimum SAR exist), we are not able to prove by theory the convergence to the maximum of the proposed algorithm (eg, using the Banach fixed‐point theorem). However, even if strictly speaking this is not true (because this is not a gradient ascent method), it can be observed that, at each iteration, the defined $G\left(\phi \right)$ function updates the solution “following” the direction of the gradient $\nabla SAR\left(\phi \right)$. Thus, the minimum SAR corresponds to an unstable fixed point.

The numerical results also experimentally demonstrate that it always converges towards the fixed point corresponding to the maximum. Indeed, starting from any initial phase set $\phi^{0}$, the phase set $\phi_{\mathrm{TM}}$ that maximized the SAR for each voxel is obtained in a few iterative steps.

Furthermore, it is also worth mentioning that the time‐dependent drive vector of sophisticated RF pulses design strategies (eg, SPINS^5^ or *k*_T_‐points^6^) produces a time‐dependent SAR distribution with the peak value in a different location for each time‐step. Considering the amplitude setting at each time‐step, the proposed method can be used to determine the worst‐case SAR for every $Q_{10g}$ matrices (or VOPs). Subsequently, the worst‐case SAR values of each $Q_{10g}$ matrix can be integrated over time and the overall worst‐case pSAR value can be assessed (Supporting Information Figure S5).

Finally, the small set of additional results confirm the validity of the assumption made for the two worst‐case pSAR approximations. One dominant eigenvalue generally exists in those regions where the maximum pSAR is located (Supporting Information Figures S6‐S8).

Indeed, the proposed bounds are very tight making them effectively quite accurate approximations of worst‐case pSAR. However, these approximations are not equally accurate. For a given amplitude set, the worst‐case pSAR approximation based on the eigenvector phase (lower‐bound) is one order of magnitude more accurate than the worst‐case pSAR approximation based on the sum of the magnitude terms only (upper‐bound).

## 6 CONCLUSIONS

In this work, a TM method for actual worst‐case pSAR determination without phase monitoring is presented. This maximization method takes advantage of the sinusoidal relation between the SAR in each voxel and the phases of input signals, to return the maximum achievable SAR for each voxel in less than 0.1 ms.

In addition to the TM method, in this work are also presented two approximations of the actual worst‐case pSAR which can be calculated almost two orders of magnitude faster than the actual worst‐case pSAR. The results show that both approximation methods are highly accurate with a mean estimation error ranging from 0.01% to 0.4%, although a considerable estimation error can rarely occur (up to 8%).

## CONFLICT OF INTEREST

Mr. Meliadò is an employee of Tesla Dynamic Coils.

## Supporting information


**FIGURE S1** Sinusoidal relation between SAR in a voxel and drive vector phases in case of: 2 (first row), 3 (second row), and 4 (third row) transmit channels. The local maximum values are periodic repetitions of the global maximum and are found on parallel “straight” multidimensional lines
**FIGURE S2** Body transmit array with 8 fractionated dipoles. Transverse and coronal sections of the ratio between each E‐field component and maximum achievable E‐field for each voxel (eg, $\sum_{i=1}^{N_{c}}\left|E_{x,i}\right|/\sum_{i=1}^{N_{c}}\left|E_{i}\right|$, where $N_{c}$ is the number of channels)
**FIGURE S3** Head transmit array with 8 oblique fractionated dipoles. Transverse and sagittal sections of the ratio between each E‐field component and maximum achievable E‐field for each voxel (eg, $\sum_{i=1}^{N_{c}}\left|E_{x,i}\right|/\sum_{i=1}^{N_{c}}\left|E_{i}\right|$, where $N_{c}$ is the number of channels)
**FIGURE S4** Head transmit array with 8 rectangular loops. Transverse and sagittal sections of the ratio between each E‐field component and maximum achievable E‐field for each voxel (eg, $\sum_{i=1}^{N_{c}}\left|E_{x,i}\right|/\sum_{i=1}^{N_{c}}\left|E_{i}\right|$, where $N_{c}$ is the number of channels)
**FIGURE S5** Worst‐case SAR distribution for sophisticated RF pulses design strategies (eg, SPINS RF pulses for body transmission array with 8 fractionated dipoles). (A) Instantaneous power of SPINS RF pulses (1 W average power limit per channel). (B) Transverse maximum intensity projection of the worst‐case SAR distributions with SPINS RF pulses
**FIGURE S6** Body transmission array with 8 fractionated dipoles. Transverse and coronal sections of the distribution of the largest eigenvalue of 10g averaged Q‐Matrices ($\lambda_{\max}$) and the ratio of second and third eigenvalues with it. The hot spots in the first column (high eigenvalues) show the regions where peak local SAR values are usually located. The second and third columns show as the second ($\lambda_{2}$) and third ($\lambda_{3}$) eigenvalues are usually very much lower in those regions
**FIGURE S7** Head transmit array with 8 oblique fractionated dipoles. Transverse and sagittal sections of the distribution of the largest eigenvalue of 10g averaged Q‐Matrices ($\lambda_{\max}$) and the ratio of second and third eigenvalues with it. The hot spots in the first column (high eigenvalues) show the regions where peak local SAR values are usually located. The second and third columns show as the second ($\lambda_{2}$) and third ($\lambda_{3}$) eigenvalues usually are very much lower in those regions
**FIGURE S8** Head transmit array with 8 rectangular loops. Transverse and sagittal sections of the distribution of the largest eigenvalue of 10g averaged Q‐Matrices ($\lambda_{\max}$) and the ratio of second and third eigenvalues with it. The hot spots in the first column (high eigenvalues) show the regions where peak local SAR values are usually located. The second and third columns show as the second ($\lambda_{2}$) and third ($\lambda_{3}$) eigenvalues are usually very much lower in those regions
**TABLE S1** Correction factors for the latest published reference‐phases‐based method to approximate the maximum achievable pSAR for each array setup

## APPENDIX 1

To define this $G\left(\phi \right)$ function, we transform the system of stationary equations $\nabla SAR\left(\phi \right)=0$. Continuing from Equations (16) and (17), using again the addition and subtraction formulas for sine and cosine, the Equation (16) becomes:

(A1.1) $$\begin{array}{c} \sum_{m=l+1}^{N_{c}}Ay_{l,m}\text{cos}\left(\phi_{l}\right)\text{cos}\left(\phi_{m}\right)+Ay_{l,m}\text{sin}\left(\phi_{l}\right)\text{sin}\left(\phi_{m}\right)‐Ax_{l,m}\text{sin}\left(\phi_{l}\right)\text{cos}\left(\phi_{m}\right)+Ax_{l,m}\text{cos}\left(\phi_{l}\right)\text{sin}\left(\phi_{m}\right)\\ ‐\sum_{n=1}^{l‐1}Ay_{n,l}\text{cos}\left(\phi_{n}\right)\text{cos}\left(\phi_{l}\right)+Ay_{n,l}\text{sin}\left(\phi_{n}\right)\text{sin}\left(\phi_{l}\right)‐Ax_{n,l}\text{sin}\left(\phi_{n}\right)\text{cos}\left(\phi_{l}\right)+Ax_{n,l}\text{cos}\left(\phi_{n}\right)\text{sin}\left(\phi_{l}\right)=0. \end{array}$$

Then, bringing out $\text{sin}\left(\phi_{l}\right)$ and $\text{cos}\left(\phi_{l}\right)$ and bringing the term with $\text{cos}\left(\phi_{l}\right)$ on the other side of the equation results in:

(A1.2) $$‐\text{sin}\left(\phi_{l}\right)\left(\sum_{m=l+1}^{N_{c}}Ay_{l,m}\text{sin}\left(\phi_{m}\right)‐Ax_{l,m}\text{cos}\left(\phi_{m}\right)‐\sum_{n=1}^{l‐1}Ay_{n,l}\text{sin}\left(\phi_{n}\right)+Ax_{n,l}\text{cos}\left(\phi_{n}\right)\right)=\text{cos}\left(\phi_{l}\right)\left(\sum_{m=l+1}^{\mathrm{Nc}}Ay_{l,m}\text{cos}\left(\phi_{m}\right)+Ax_{l,m}\text{sin}\left(\phi_{m}\right)‐\sum_{n=1}^{l‐1}Ay_{n,l}\text{cos}\left(\phi_{n}\right)‐Ax_{n,l}\text{sin}\left(\phi_{n}\right)\right).$$

From this, the expression of the tangent of $\phi_{l}$ can be obtained

(A1.3) $$\text{tan}\left(\phi_{l}\right)=\frac{\text{sin}\left(\phi_{l}\right)}{\text{cos}\left(\phi_{l}\right)}=\frac{\left(\sum_{m=l+1}^{N_{c}}Ay_{l,m}\text{cos}\left(\phi_{m}\right)+Ax_{l,m}\text{sin}\left(\phi_{m}\right)‐\sum_{n=1}^{l‐1}Ay_{n,l}\text{cos}\left(\phi_{n}\right)‐Ax_{n,l}\text{sin}\left(\phi_{n}\right)\right)}{‐\left(\sum_{m=l+1}^{N_{c}}Ay_{l,m}\text{sin}\left(\phi_{m}\right)‐Ax_{l,m}\text{cos}\left(\phi_{m}\right)‐\sum_{n=1}^{l‐1}Ay_{n,l}\text{sin}\left(\phi_{n}\right)+Ax_{n,l}\text{cos}\left(\phi_{n}\right)\right)}.$$

Hence, the $\phi_{l}$ can be determined by knowing the other phases

(A1.4) $$\phi_{l}=\text{arctan}\left(\frac{\left(\sum_{m=l+1}^{N_{c}}Ay_{l,m}\text{cos}\left(\phi_{m}\right)+Ax_{l,m}\text{sin}\left(\phi_{m}\right)‐\sum_{n=1}^{l‐1}Ay_{n,l}\text{cos}\left(\phi_{n}\right)‐Ax_{n,l}\text{sin}\left(\phi_{n}\right)\right)}{‐\left(\sum_{m=l+1}^{N_{c}}Ay_{l,m}\text{sin}\left(\phi_{m}\right)‐Ax_{l,m}\text{cos}\left(\phi_{m}\right)‐\sum_{n=1}^{l‐1}Ay_{n,l}\text{sin}\left(\phi_{n}\right)+Ax_{n,l}\text{cos}\left(\phi_{n}\right)\right)}\right).$$
