# Supplementary material for: Real‐time assessment of potential peak local specific absorption rate value without phase monitoring: Trigonometric maximization method for worst‐case local specific absorption rate determination
Source: Magn Reson Med. 2020 Dec 22;85(6):3420–33. doi: 10.1002/mrm.28635 (PMC7986921; doi:10.1002/mrm.28635)
Supplement: Supplementary file 1 — FIGURE S1 Sinusoidal relation between SAR in a voxel and drive vector phases in case of: 2 (first row), 3 (second row), and 4 (third row) transmit channels. The local maximum values are periodic repetitions of the global maximum and are found on parallel “straight” multidimensional lines FIGURE S2 Body transmit array with 8 fractionated dipoles. Transverse and coronal sections of the ratio between each E‐field component and maximum achievable E‐field for each voxel (eg, ∑i=1NcEx,i/∑i=1NcEi, where Nc is the number of channels) FIGURE S3 Head transmit array with 8 oblique fractionated dipoles. Transverse and sagittal sections of the ratio between each E‐field component and maximum achievable E‐field for each voxel (eg, ∑i=1NcEx,i/∑i=1NcEi, where Nc is the number of channels) FIGURE S4 Head transmit array with 8 rectangular loops. Transverse and sagittal sections of the ratio between each E‐field component and maximum achievable E‐field for each voxel (eg, ∑i=1NcEx,i/∑i=1NcEi, where Nc is the number of channels) FIGURE S5 Worst‐case SAR distribution for sophisticated RF pulses design strategies (eg, SPINS RF pulses for body transmission array with 8 fractionated dipoles). (A) Instantaneous power of SPINS RF pulses (1 W average power limit per channel). (B) Transverse maximum intensity projection of the worst‐case SAR distributions with SPINS RF pulses FIGURE S6 Body transmission array with 8 fractionated dipoles. Transverse and coronal sections of the distribution of the largest eigenvalue of 10g averaged Q‐Matrices (λmax) and the ratio of second and third eigenvalues with it. The hot spots in the first column (high eigenvalues) show the regions where peak local SAR values are usually located. The second and third columns show as the second (λ2) and third (λ3) eigenvalues are usually very much lower in those regions FIGURE S7 Head transmit array with 8 oblique fractionated dipoles. Transverse and sagittal sections of the distribution of the largest eigenvalue of 10g av [file MRM-85-3420-s001.docx]

**Supporting Information:**

**Real-time assessment of potential peak local SAR value without phase monitoring: Trigonometric maximization method for worst-case local SAR determination**

E.F. Meliadò, A. Sbrizzi, C.A.T van den Berg, P.R. Luijten, and A.J.E Raaijmakers

Correction factors:

| **Reference**  **Phase Set**  $\boldsymbol{P}_{k}$ | **Correction Factors** $\zeta_{k}$ | | |
| --- | --- | --- | --- |
|  | **Body Array**  **(8 Fractionated Dipoles)** | **Head Array**  **(8 Fractionated Dipoles)** | **Head Array**  **(8 Rectangular Loops)** |
| $k=1$ | 2.59 | 7.58 | 3.79 |
| $k=2$ | 2.87 | 7.59 | 3.49 |
| $k=3$ | 2.62 | 4.54 | 2.52 |
| $k=4$ | 2.58 | 3.81 | 4.43 |
| $k=5$ | 2.82 | 6.06 | 6.09 |
| $k=6$ | 2.68 | 3.82 | 4.34 |
| $k=7$ | 2.61 | 5.65 | 2.72 |
| $k=8$ | 2.95 | 7.78 | 3.42 |

**Supporting Information Table S1:** Correction factors for the latest published reference-phases-based method to approximate the maximum achievable pSAR for each array setup.

Sinusoidal relation between SAR and drive vector phases.


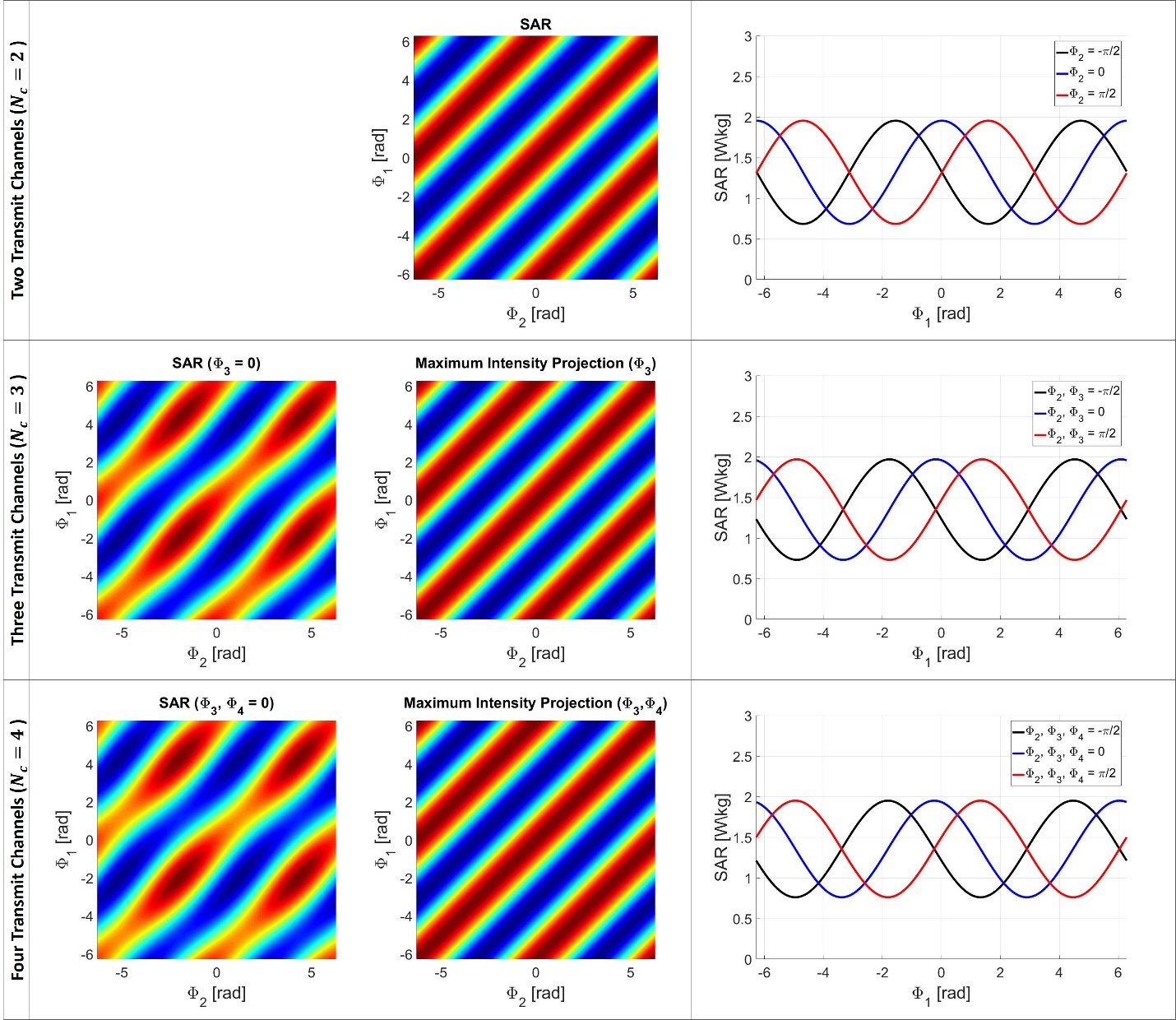


**Supporting Information Figure S1:** Sinusoidal relation between SAR in a voxel and drive vector phases in case of: 2 (first row), 3 (second row), and 4 (third row) transmit channels. The local maximum values ​​are periodic repetitions of the global maximum and are found on parallel "straight" multidimensional lines.

Dominant z-component of the transmitted E-field: Body transmit array with 8 fractionated dipoles.


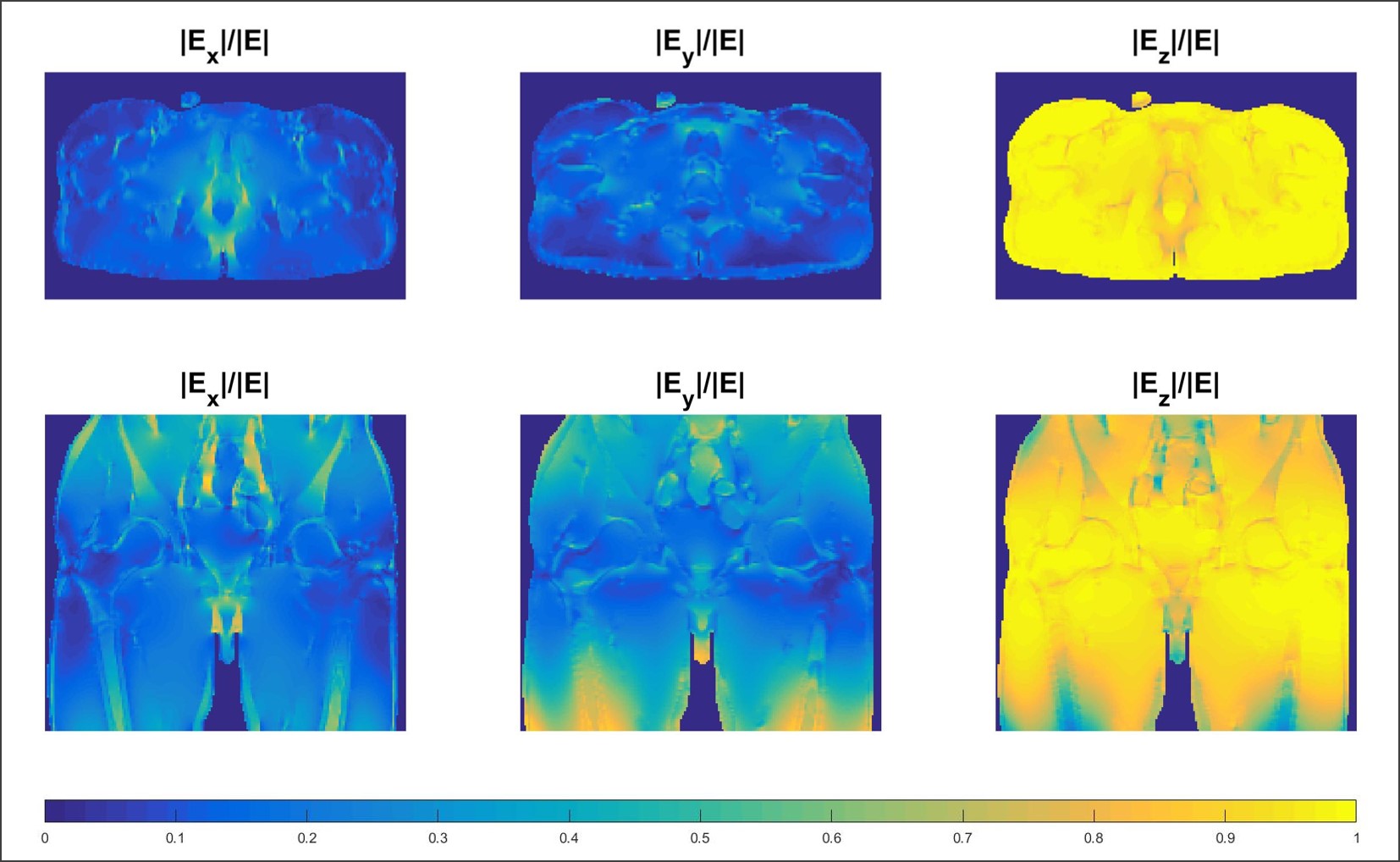


**Supporting Information Figure S2:** Body transmit array with 8 fractionated dipoles. Transverse and coronal sections of the ratio between each E-field component and maximum achievable E-field for each voxel (e.g. ${\sum_{i=1}^{N_{c}} \left| E_{x,i} \right|}/{\sum_{i=1}^{N_{c}} \left| E_{i} \right|}$, where $N_{c}$ is the number of channels).

Dominant z-component of the transmitted E-field: Head transmit array with 8 fractionated dipoles.

**
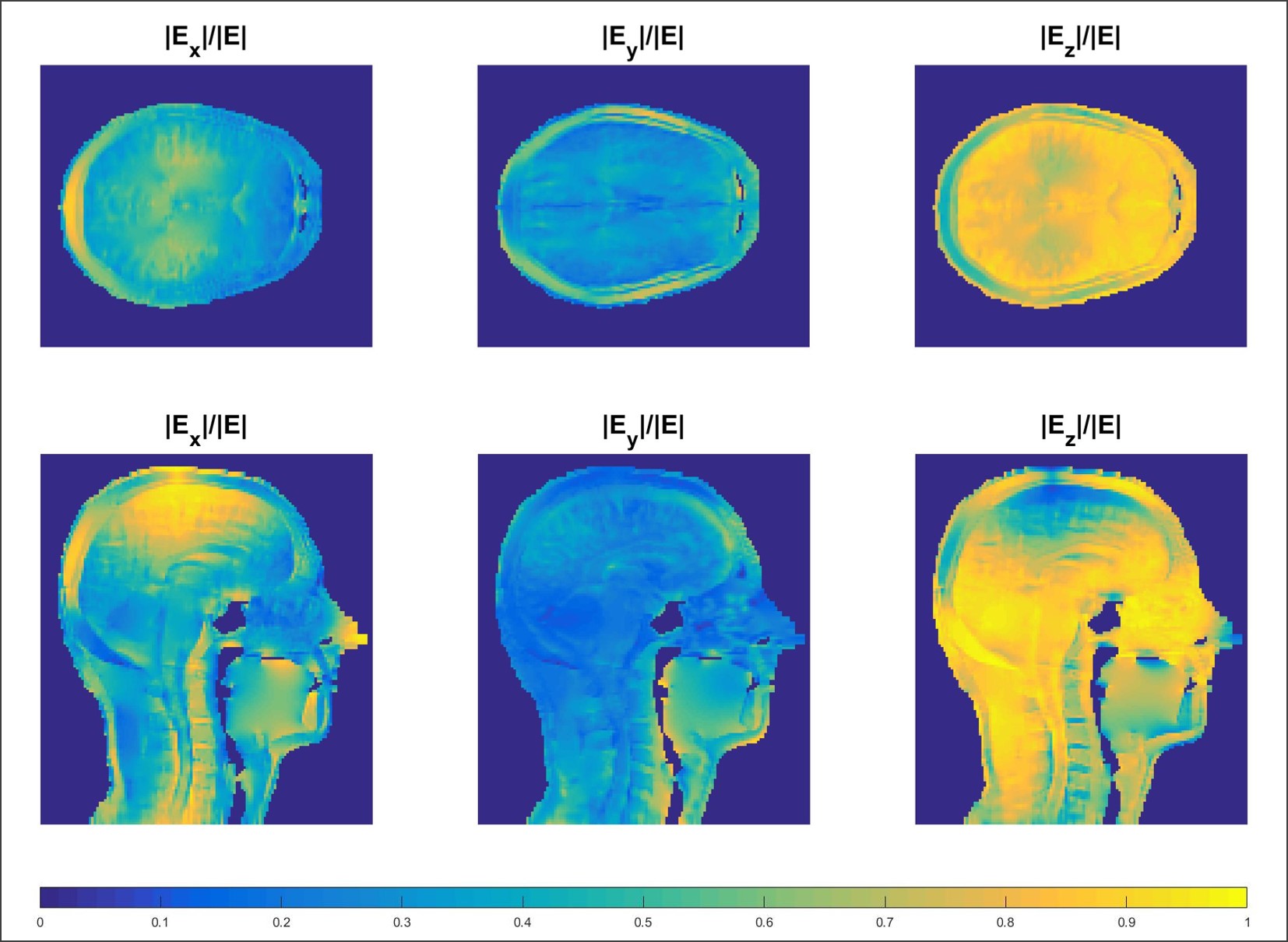
**

**Supporting Information Figure S3:** Head transmit array with 8 oblique fractionated dipoles. Transverse and sagittal sections of the ratio between each E-field component and maximum achievable E-field for each voxel (e.g. ${\sum_{i=1}^{N_{c}} \left| E_{x,i} \right|}/{\sum_{i=1}^{N_{c}} \left| E_{i} \right|}$, where $N_{c}$ is the number of channels).

Dominant z-component of the transmitted E-field: Head transmit array with 8 rectangular loops.


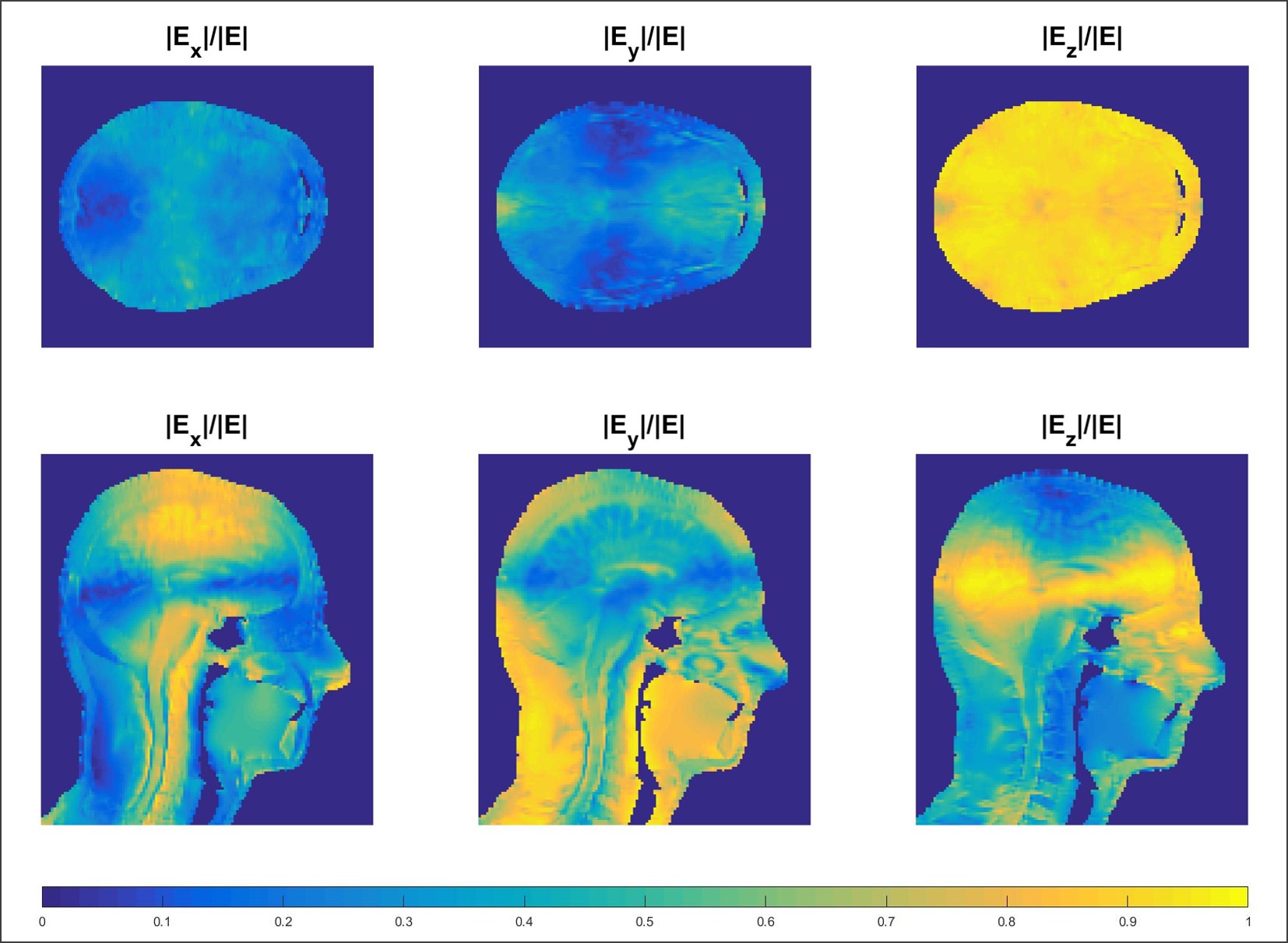


**Supporting Information Figure S4:** Head transmit array with 8 rectangular loops. Transverse and sagittal sections of the ratio between each E-field component and maximum achievable E-field for each voxel (e.g. ${\sum_{i=1}^{N_{c}} \left| E_{x,i} \right|}/{\sum_{i=1}^{N_{c}} \left| E_{i} \right|}$, where $N_{c}$ is the number of channels).

Worst-Case SAR Distribution for time-dependent RF pulses.


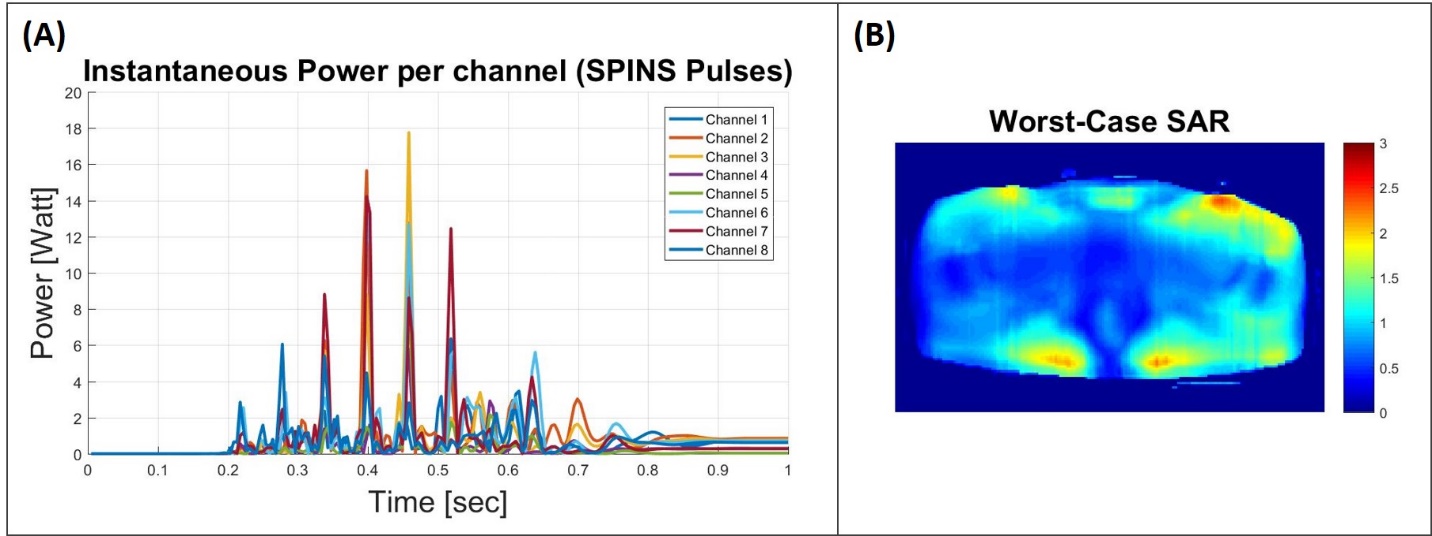


**Supporting Information Figure S5:** Worst-case SAR distribution for sophisticated RF pulses design strategies (e.g. SPINS RF pulses for body transmission array with 8 fractionated dipoles). (A) Instantaneous power of SPINS RF pulses (1 W average power limit per channel). (B) Transverse maximum intensity projection of the worst-case SAR distributions with SPINS RF pulses.

Dominant Eigenvalue: Body transmit array with 8 fractionated dipoles.


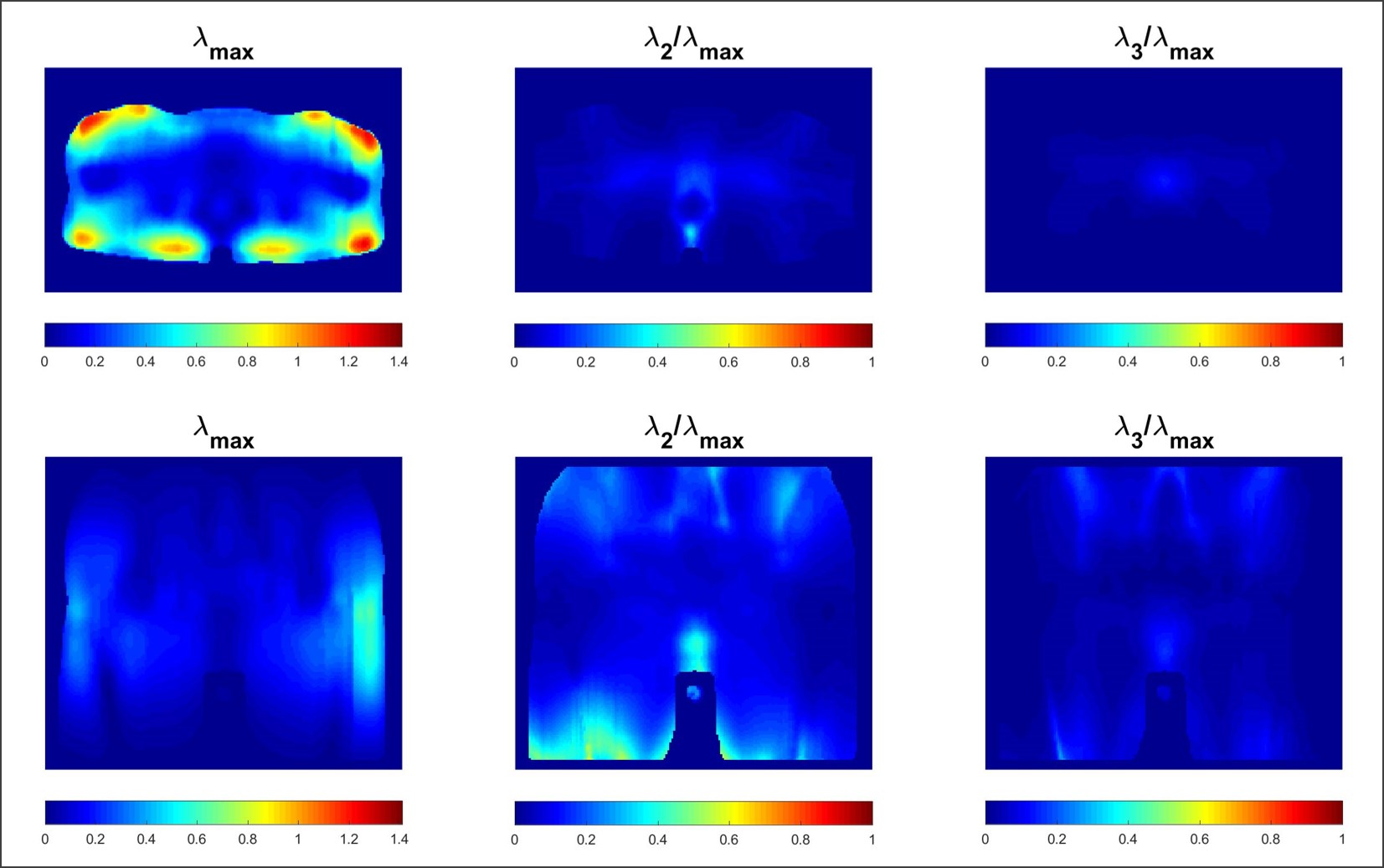


**Supporting Information Figure S6:** Body transmission array with 8 fractionated dipoles. Transverse and coronal sections of the distribution of the largest eigenvalue of 10g averaged Q-Matrices ($\lambda_{max}$) ​​and the ratio of second and third eigenvalues ​​with it. The hot spots in the first column (high eigenvalues) show the regions where peak local SAR values are usually located. The second and third columns show as the second ($\lambda_{2}$) and third ($\lambda_{3}$) eigenvalues are usually very much lower in those regions.

Dominant Eigenvalue: Head transmit array with 8 fractionated dipoles.


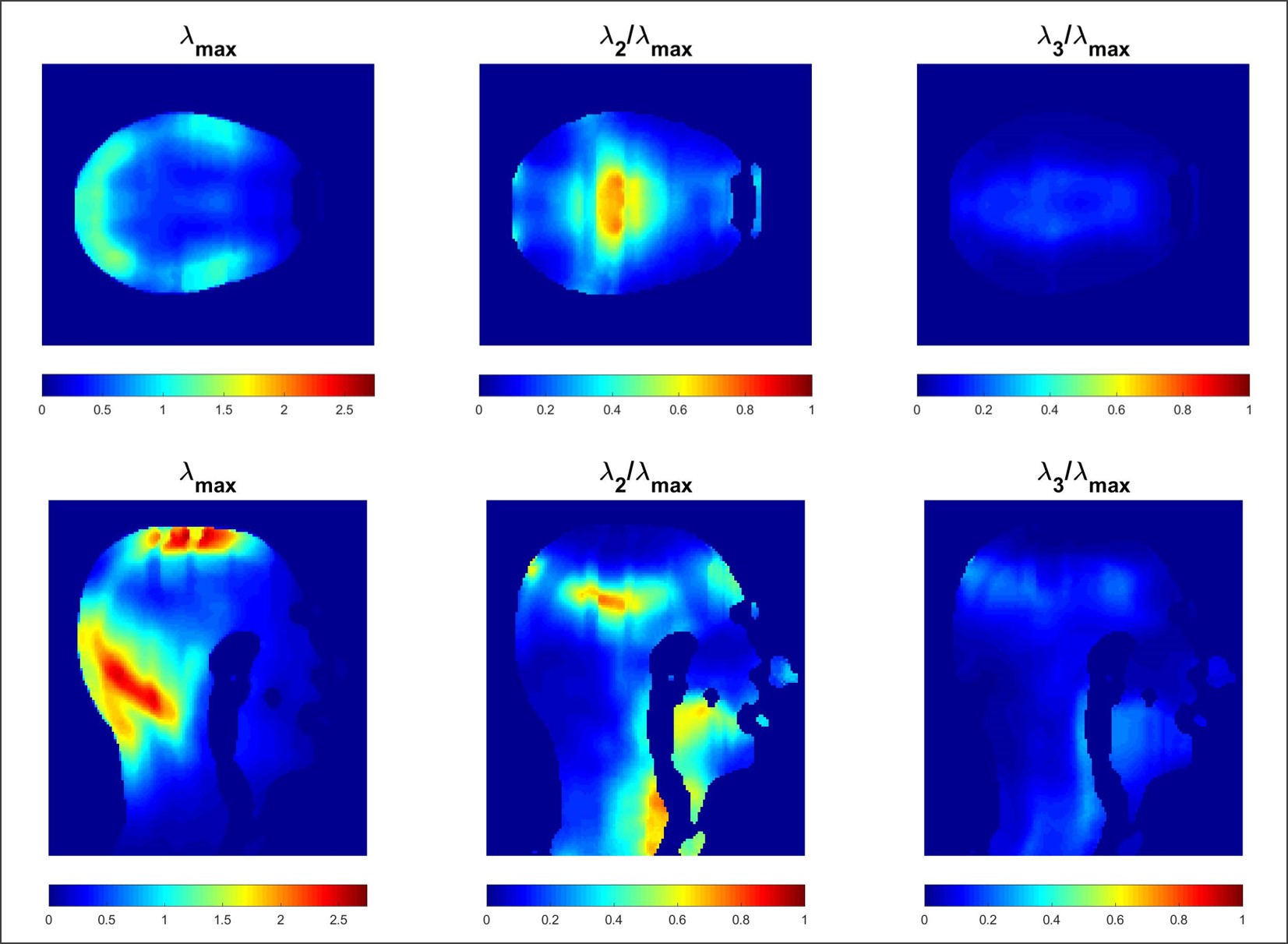


**Supporting Information Figure S7:** Head transmit array with 8 oblique fractionated dipoles. Transverse and sagittal sections of the distribution of the largest eigenvalue of 10g averaged Q-Matrices ($\lambda_{max}$) ​​and the ratio of second and third eigenvalues ​​with it. The hot spots in the first column (high eigenvalues) show the regions where peak local SAR values are usually located. The second and third columns show as the second ($\lambda_{2}$) and third ($\lambda_{3}$) eigenvalues usually are very much lower in those regions.

Dominant Eigenvalue: Head transmit array with 8 rectangular loops.


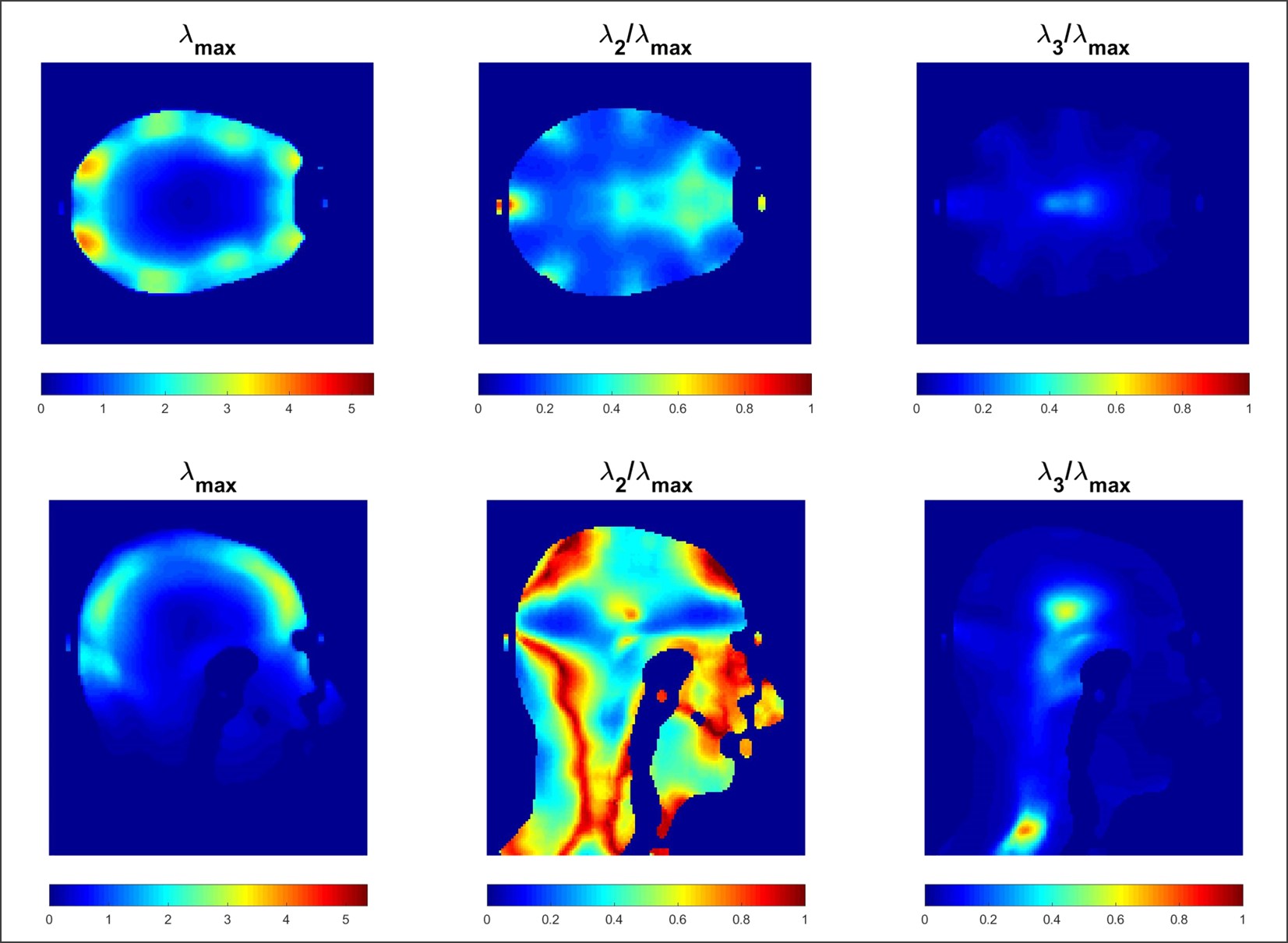


**Supporting Information Figure S8:** Head transmit array with 8 rectangular loops. Transverse and sagittal sections of the distribution of the largest eigenvalue of 10g averaged Q-Matrices ($\lambda_{max}$) ​​and the ratio of second and third eigenvalues ​​with it. The hot spots in the first column (high eigenvalues) show the regions where peak local SAR values are usually located. The second and third columns show as the second ($\lambda_{2}$) and third ($\lambda_{3}$) eigenvalues are usually very much lower in those regions.

**APPENDIX S1**

Suppose that only the z-component of the E-fields are present, then:

| $\boldsymbol{Q}=\frac{\sigma}{2\rho}\left( {\tilde{\boldsymbol{E}}}_{z} \right)^{\dagger}{\tilde{\boldsymbol{E}}}_{z}$ | [S1.1] |
| --- | --- |

Following the Eigenvalue equation:

| $\boldsymbol{Qv}=\lambda\boldsymbol{v}\frac{\sigma}{2\rho}\left( {\tilde{\boldsymbol{E}}}_{z} \right)^{\dagger}{\tilde{\boldsymbol{E}}}_{z} \boldsymbol{v=}\lambda\boldsymbol{v}$ | [S1.2] |
| --- | --- |

Setting

| $\boldsymbol{v=}\frac{\left( {\tilde{\boldsymbol{E}}}_{z} \right)^{\dagger}}{\left\Vert{\tilde{\boldsymbol{E}}}_{z} \right\Vert}$ | [S1.3] |
| --- | --- |

and substituting

| $\frac{\sigma}{2\rho}\left( {\tilde{\boldsymbol{E}}}_{z} \right)^{\dagger}{\tilde{\boldsymbol{E}}}_{z}{\frac{\left( {\tilde{\boldsymbol{E}}}_{z} \right)^{\dagger}}{\left\Vert{\tilde{\boldsymbol{E}}}_{z} \right\Vert}}_{z}\boldsymbol{=}\lambda\frac{\left( {\tilde{\boldsymbol{E}}}_{z} \right)^{\dagger}}{\left\Vert{\tilde{\boldsymbol{E}}}_{z} \right\Vert} \underset{\Rightarrow}{}\lambda=\frac{\sigma}{2\rho}\left\Vert{\tilde{\boldsymbol{E}}}_{z} \right\Vert^{2}$ | [S1.4] |
| --- | --- |

Then, ${\left( {\tilde{\boldsymbol{E}}}_{z} \right)^{\dagger}}/\left\| {\tilde{\boldsymbol{E}}}_{z} \right\|$ is the only eigenvector of $\boldsymbol{Q}$ with non-zero eigenvalue given by $\lambda$ as above.
